# More Favorable Palmitic Acid Over Palmitoleic Acid Modification of Wnt3 Ensures Its Localization and Activity in Plasma Membrane Domains

**DOI:** 10.3389/fcell.2019.00281

**Published:** 2019-11-15

**Authors:** Yagmur Azbazdar, Ozgun Ozalp, Erdinc Sezgin, Sapthaswaran Veerapathiran, Anna L. Duncan, Mark S. P. Sansom, Christian Eggeling, Thorsten Wohland, Ezgi Karaca, Gunes Ozhan

**Affiliations:** ^1^Izmir Biomedicine and Genome Center (IBG), Dokuz Eylul University Health Campus, Inciralti-Balcova, Izmir, Turkey; ^2^Izmir International Biomedicine and Genome Institute (IBG-Izmir), Dokuz Eylul University, Inciralti-Balcova, Izmir, Turkey; ^3^MRC Human Immunology Unit, Weatherall Institute of Molecular Medicine, University of Oxford, Oxford, United Kingdom; ^4^Department of Biological Sciences and Center for BioImaging Sciences, National University of Singapore, Singapore, Singapore; ^5^Department of Biochemistry, University of Oxford, Oxford, United Kingdom; ^6^Department of Super-Resolution Microscopy, Institute for Applied Optics and Biophysics, Friedrich-Schiller-University Jena, Jena, Germany; ^7^Department of Biophysical Imaging, Leibniz Institute of Photonic Technology e.V., Jena, Germany; ^8^Department of Chemistry, National University of Singapore, Singapore, Singapore

**Keywords:** ordered plasma membrane domain, lipid raft, Wnt/β-catenin pathway, structural modeling, acylation, palmitoylation

## Abstract

While the lateral organization of plasma membrane components has been shown to control binding of Wnt ligands to their receptors preferentially in the ordered membrane domains, the role of posttranslational lipid modification of Wnt on this selective binding is unknown. Here, we identify that the canonical Wnt is presumably acylated by palmitic acid, a saturated 16-carbon fatty acid, at a conserved serine residue. Acylation of Wnt3 is dispensable for its secretion and binding to Fz8 while it is essential for Wnt3's proper binding and domain-like diffusion in the ordered membrane domains. We further unravel that non-palmitoylated Wnt3 is unable to activate Wnt/β-catenin signaling either in zebrafish embryos or in mammalian cells. Based on these results, we propose that the lipidation of canonical Wnt, presumably by a saturated fatty acid, determines its competence in interacting with the receptors in the appropriate domains of the plasma membrane, ultimately keeping the signaling activity under control.

## Introduction

Wnt/β-catenin signaling, the so-called canonical Wnt pathway, regulates a broad range of biological processes during embryonic development, adult tissue homeostasis and tissue regeneration (Huang and He, 2008; Clevers and Nusse, 2012; Ozhan and Weidinger, 2014). Aberrant Wnt signaling is related to various types of cancer, congenital defects and degenerative diseases (Logan and Nusse, 2004; Clevers, 2006; Nusse and Clevers, 2017). Wnts are lipid- and sugar-modified morphogens that play critical roles in cells through engagement of a receptor complex that includes Frizzled (Fz) and low-density lipoprotein receptor-related protein 5/6 (LRP 5/6). Wnt/β-catenin signaling is kept in the OFF-state in the absence of an active canonical Wnt ligand, leading to phosphorylation of β-catenin in the cytoplasm by the destruction complex and its proteasomal degradation (Angers and Moon, 2009; MacDonald et al., 2009). Transition from the Wnt-OFF- to the Wnt-ON-state is initiated by the binding of a canonical Wnt ligand to a canonical Fz receptor and the co-receptor low-density lipoprotein receptor-related protein 5/6 (Lrp5/6) (Angers and Moon, 2009). The formation of Wnt-receptor complex triggers a series of cellular events that include phosphorylation and endocytosis of Lrp5/6, recruitment of the cytoplasmic proteins Disheveled (Dvl) and Axin to the receptor complex, inhibition of the destruction complex, stabilization of cytoplasmic β-catenin and translocation of stabilized β-catenin to the nucleus where it interacts with the transcription factors of the lymphoid enhancer-binding factor (Lef) and T cell factor (Tcf) family to activate gene expression (Kikuchi and Yamamoto, 2007; Yamamoto et al., 2008; Niehrs and Shen, 2010).

A key step underlying initiation of the pathway is the binding of canonical Wnt ligands to their receptor complexes at the plasma membrane. The plasma membrane contains ordered membrane domains, conventionally referred to as membrane (lipid) rafts, that are highly dynamic membrane regions characterized by the selective recruitment of saturated lipids, sterols and specific lipid-anchored proteins (Simons and Ikonen, 1997; Sezgin et al., 2017b). These ordered structures generate compact transient platforms for ligand-receptor interaction and receptor clustering, and are critical in signal transduction pathways including the canonical Wnt signaling (Simons and Toomre, 2000; Jury et al., 2007; Midgley et al., 2013; Ozhan et al., 2013; Dinic et al., 2015; Guven-Maiorov et al., 2015; Sezgin et al., 2017b; Agarwal et al., 2018; Badawy et al., 2018). In contrast to the scattered distribution of the canonical pathway receptor Fz8 and the coreceptor Lrp6 throughout the membrane, the membrane-bound Wnt pathway modulator Lypd6 becomes localized to the ordered membrane domains and ensures via direct physical interaction that Lrp6 is phosphorylated in these domains to activate signaling (Yamamoto et al., 2008; Sakane et al., 2010; Ozhan et al., 2013). Our recent work has unraveled the influence of the immediate plasma membrane environment on the canonical Wnt–receptor interaction by showing that canonical Wnt selectively binds to its pool of receptors in the ordered domains and this domain-specific binding is necessary for downstream signaling activity (Sezgin et al., 2017b). Nevertheless, the link between lipid modifications of Wnt and its preferential binding to specific membrane regions has remained elusive.

Wnt proteins are extensively modified through glycosylation and acylation at the post-translational level. Mutations introduced at N-linked glycosylation sites of different Wnt proteins suggest that glycosylation plays critical roles in Wnt folding and subsequent secretion (Mason et al., 1992; Komekado et al., 2007; Kurayoshi et al., 2007). In contrast to glycosylation, acylation is essential for Wnt activity (Willert and Nusse, 2012). Initially, mass spectrometric analysis of the purified mouse Wnt3a (mWnt3a) exhibited two different acyl group modifications, i.e., a thioester-linked palmitic acid at a conserved cysteine (C77 in murine Wnt3a) and an oxyester-linked palmitoleic acid at a conserved serine (S209 in murine Wnt3a) (Willert et al., 2003; Takada et al., 2006). In the later published high-resolution structure of *Xenopus* Wnt8 (xWnt8) in complex with the extracellular cysteine-rich domain (CRD) of mouse Fz8 (Protein Data Bank [PDB] id: 4F0A), the conserved serine was found to be acylated, suggesting this serine as a consensus acylation site across all Wnts (Janda et al., 2012; Willert and Nusse, 2012). In this study, the chemical identity of the lipid linked to xWnt8 could not be unambiguously detected by mass spectrometry (Janda et al., 2012).

The role of lipid modifications in secretion and functionality of Wnt has also been studied in several Wnt proteins by mutating conserved acylation sites (Kurayoshi et al., 2007; Franch-Marro et al., 2008; Tang et al., 2012; Luz et al., 2014). Mutagenesis of the conserved serine Wnt acylation sites (S209 in mWnt3a and S239 in *Drosophila* Wingless [Wg]) consistently abolished Wnt function in different species (Takada et al., 2006; Franch-Marro et al., 2008). Yet, the amount of Wnt secreted from mWnt3aS209A mutant was dramatically reduced when compared to Wnt secreted from WgS239A mutant. Mutation introduced at the conserved serine residue of zebrafish Wnt8a reduced both its secretion and signaling capability (Luz et al., 2014). On the contrary, mWnt1 and mWnt3a without any lipid adducts were secreted but non-functional (Doubravska et al., 2011). Therefore, the impact of acylation on the secretion of different types of Wnts is still a matter of debate.

Another ambiguity in the literature emanates from the influence of acylation on protein binding to the plasma membrane. S acylation typically by a saturated 16-carbon fatty acid, i.e., palmitoylation, determines the ability of soluble proteins to associate with the membrane and membrane-associated proteins and targets them into ordered membrane domains, suggesting a link between order preference and signaling activation (Levental et al., 2010). Thus, acylation of a canonical Wnt ligand by a monounsaturated fatty acid (Takada et al., 2006) appears to strongly contradict Wnt's membrane binding and activation of signaling preferentially in the ordered domains (Zhai et al., 2004; Ozhan et al., 2013; Sezgin et al., 2017a).

To address all the above-mentioned open questions, here we address the type and influence of the lipid modification on the ability of canonical Wnt ligand in binding to the plasma membrane and activation of Wnt/β-catenin signaling. To this end, we have conformationally analyzed the accessible palmitoleic acid (PAM; 16:1) and palmitic acid (PLM, 16:0) structures (deposited in the PDB). Upon comparing the acquired PAM/PLM coordinates with the fatty acid present in the crystal structure of xWnt8-mouse:Fz8-CRD, we have shown that the fatty acid molecule within the xWnt8-mouse:Fz8-CRD complex is conformationally closest to a PLM molecule bound to the human acyloxyacyl hydrolase (PDB id: 5W78). Based on this, we have constructed the atomistic models of Fz8-CRD bound to PAM and PLM, which has demonstrated that only Wnt8's acylation by PLM is conformationally permissive. To further investigate the functional role of acylation in zebrafish Wnt3, a canonical Wnt ligand, we have generated a point mutation in the conserved serine at position 212, namely S212 (homologous to S209 in mWnt3a). Our data have shown that this specific acylation of Wnt3 ligand is not essential for its secretion and physical interaction with its receptor Fz8 at the plasma membrane. It is, however, required for Wnt's localization to the ordered domains of the plasma membrane, where the ligand selectively binds to its receptors and co-internalizes with the receptor complex. Using Imaging Total Internal Reflection Fluorescence Correlation Spectroscopy (ITIR-FCS) diffusion law, we have demonstrated that acylation is indispensable for the domain-like diffusion of Wnt3 in the ordered membrane regions, and for Wnt3 to activate canonical Wnt signaling in both zebrafish embryos and mammalian cells. In the light of these findings, here we propose that the acylation of Wnt3 ligand with a saturated palmitic acid ensures its partitioning into the ordered membrane domains and the downstream canonical Wnt signaling activity. Overall, by using a prominent combination of computational and experimental work, we underscore the significance of Wnt acylation, presumably by a palmitic acid, in receptor targeting within the ordered domains and subsequent signaling activation.

## Materials and Methods

### Analysis of the Available PAM/PLM Conformations

**Figure 1:**
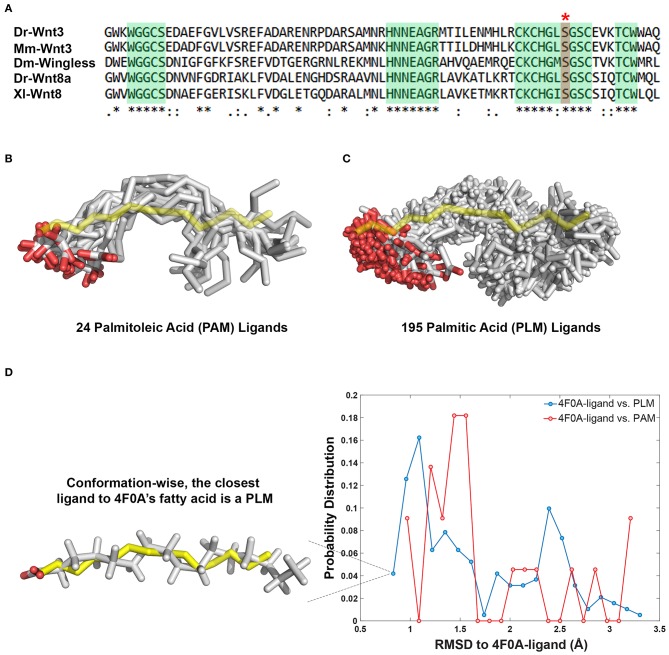
PAM and PLM can adopt a diverse range of conformations. **(A)** Protein multiple sequence alignment covering a region conserved between two canonical Wnt ligands (Wnt3 and Wnt8) of vertebrates and Wingless (Wg) of *Drosophila*. A conserved serine amino acid (red marked column with asterisk) has been shown to be acylated in various ligands. Consensus sequences are marked in green. Dr *Danio rerio*, Mm, *Mus musculus*, Dm, *Drosophila melanogaster*, and Xl *Xenopus laevis*. **(B,C)** Structurally characterized **(B)** PAM and **(C)** PLM conformations downloaded from PDB (depicted in white sticks) aligned on the backbone of 4F0A's fatty acid (represented in yellow sticks). **(D)** The probability density distributions of being conformationally close to 4F0A's ligand. The closeness measure used is the Root-mean-square deviation (RMSD) from 4F0A's ligand, calculated over its carbon atoms. Available PLM ligand conformers have a higher probability to be <1 Å away from 4F0A's ligand. The closest ligand to 4F0A's ligand is a PLM molecule, which is depicted as an inset.

The 24 PAM and 195 PLM ligands deposited in the Protein Data Bank (https://www.rcsb.org) were aligned on the fatty acid provided within the PDB entry 4F0A (Janda et al., 2012). The alignment was carried out with the fitting program ProFit where 4F0A's fatty acid carbons were used as a reference (Martin and Porter, 2009). For each case, obtained fit distributions (expressed in Å) were binned into 20 with the standard histogram building option of Matlab (The MathWorks, 2018). To remove the statistical bias, the occurrence frequencies obtained with this analysis were normalized by the size of the data set. The normalized distributions were interpreted as the probability density for being conformationally close to 4F0A's ligand (Figure 1D).

### Modeling the Interatomic Contacts Formed Between PAM/PLM and Mouse Frizzled 8

In 4F0A, the fatty acid ligand spans Fz8-CRD's hydrophobic channel formed by GLN71, PHE72, PRO74, ILE78, MET122, TYR125, PHE127, PRO130 where the fatty acid's first carbon C1 is within <7 Å of GLN71 and TRP129. The latter amino acid secures the fatty acid covalently linked to Wnt. Taken this information as a basis, two modeling scenarios were constructed. In the first one, the optimal PAM coordinates (including its hydrogens) were obtained via ligand expo rscb (http://ligand-expo.rcsb.org, Figure S6). Then, the topology and parameter files of these ligands were obtained by PRODRG (http://prodrg1.dyndns.org/) (Schuttelkopf and van Aalten, 2004). These templates were used as a reference to add missing atoms of 4F0A's PAM within the integrative modeling platform HADDOCK (van Zundert et al., 2016). After proper addition of the missing atoms, PAM interactions with Fz8 were reconstructed under the effect of the interaction information listed above. The same procedure was repeated with the same starting structures, this time by supplying PLM topology and parameter files to define the ligand. The relevant restraint files, topologies and outcomes of the runs are provided under https://github.com/ezgikaraca/Wnt-acylation.

### Modeling the Interatomic Contacts Formed Between PLM and Zebrafish Frizzled 8

The homology model of the zebrafish Fz8 was constructed by using mouse Fz8 as a template within i-Tasser (https://zhanglab.ccmb.med.umich.edu/I-TASSER/) (Yang et al., 2015). After obtaining zebrafish Fz8 structure, its PLM interacting residues were determined by using structural comparison with the mouse Fz8, i.e., GLN66, PHE67, PRO 69, LEU70, ILE73, LEU116, MET117, TYR120, PHE122 (Figure S4). These residues were kept in contact with the relevant PLM atoms while the zebrafish Fz8-PLM interaction was refined in HADDOCK. Here the PLM topology and coordinates were taken from the previous modeling step (https://github.com/ezgikaraca/Wnt-acylation).

### Transgenic Fish Lines

Transgenic zebrafish (*Danio rerio*) line Tg(*7xTcfF-Xla.Siam*:nlsmCherry) was outcrossed to wild type zebrafish and sorted as described previously (Moro et al., 2012). This line was used as a reporter of Wnt/ß-catenin signaling activity.

### Cell Culture

HEK293T cells were grown in Dulbecco's Modified Eagle Medium (DMEM) supplemented with 10% fetal bovine serum (FBS) at 37°C in 5% (v/v) CO_2_ humidified environment. SH-SY5Y cells were obtained from ATCC (Manassas, VA, USA) and grown in DMEM/F12 supplemented with 10% FBS, 1% L-glutamine and 1% non-essential amino acids.

### Cloning of Zf wt Wnt3-GFP, Zf Wnt3S212A-GFP, and Zf FLAG-Fz8a

Total RNA was isolated from 24 h-post-fertilization (hpf) zebrafish embryos using Direct-zol RNA Kit (Zymo Research, Irvine, CA) and cDNA was synthesized with iScript reverse transcriptase (Biorad, Hercules, CA) using a 1:1 mixture of oligodT and random primers. For (-RT) controls, iScript RT was replaced with water. For zebrafish (Zf) wt Wnt3-GFP, PCR was performed using 1 μL cDNA with the forward primer 5′-GATCTCCACCATGGATTTGTACCTGGTTGGATT-3′ and the reverse primer 5′-GAATTCTTTACATGTATGTACGTCGTAGACC-3′. PCR product was digested with BglII and EcoRI and ligated into pCS2P+ vector that has EGFP. Zf Wnt3S212A-GFP was generated by site-directed mutagenesis at Zf wt Wnt3-GFP using overlap extension PCR. The following primers were used for the first round of PCR: forward1 5′-AGATCTCCACCATGGATTTGTACCTGGTTGGATT-3 with reverse1 5′-ACTTCACAGCTGCCAGCCAG-3′ and forward2 5′-CTGGCTGGCAGCTGTGAAGT-3′ with reverse2 5′ GAATTCTTTACATGTATGTACGTCGTAGACC-3′. The second round of PCR was conducted by using the purified PCR product of the first round of PCR as the template and the primers forward1 with reverse2. The purified PCR product of the second round of PCR was digested with BglII and EcoRI and ligated into pCS2P+ vector that has EGFP. Zf Fz8a was amplified with the following primers: forward 5′-AGAATTCAACCACCATGGAGTGCTACCT-3′ and reverse 5′- GGATCCTCAGACTTGGGACAAAGGC-3′. PCR product was digested with EcoRI and BamHI and ligated into p3xFLAG-CMV-7.1 vector. Successful cloning was verified by restriction digestion and agarose gel electrophoresis.

### Capped Sense mRNA Synthesis, Microinjection, and Whole-Mount *in situ* Hybridization

Capped sense RNAs of wt Wnt3-GFP and Wnt3S212A-GFP were synthesized with mMessage mMachine Kit (Thermo Fisher Scientific, Waltham, MA, USA). For DRM flotation assay and qPCR, 1 ng of mRNA was injected into one-cell zebrafish embryos and embryos were fixed at 6 hpf in 4% paraformaldehyde (PFA) dissolved in PBS overnight. For whole-mount *in situ* hybridization (WMISH), 200 pg mRNA was injected into one-cell zebrafish embryos and embryos were fixed at either 50% epiboly or 24 hpf in 4% PFA overnight. WMISH was performed with *mCherry, sp5l* or *foxg1a* and *her5* antisense RNA probes as described previously (Jowett and Lettice, 1994).

### Quantitative PCR (qPCR)

Capped sense RNA (wt Wnt3-GFP, Wnt3S212A-GFP or membrane-bound GFP as control) was injected into one-cell zebrafish embryos. RNA was isolated from injected embryos at 7 hpf using Direct-zol RNA kit and cDNA was synthesized with iScript reverse transcriptase (RT). 1:1 mixture of oligodT and random primers were used and qPCR was performed in triplicates using *rpl13a* primers for normalization to determine relative gene expression levels. qPCR was performed using GoTaq qPCR master mix (Promega, Madison, WI, USA) at Applied Biosystems 7500 Fast Real Time PCR Machine (Foster City, CA, USA). The following primers were used for qPCR: *mCherry* forward 5′GAACGGCCACGAGTTCGAGA-3′, *mCherry* reverse 5′CTTGGAGCCGTACATGAACTGAGG-3′, Zf *sp5l* forward 5′-GCTTCACGCAGGTGTGGAT-3′, Zf *sp5l* reverse 5′-TTCTGGAGATGAGCTGGGAGT-3′, Zf *rpl13a* forward 5′-TCTGGAGGACTGTAAGAGGTATGC-3′ and Zf *rpl13a* reverse 5′-AGACGCACAATCTTGAGAGCAG-3′.

### Transfection and Luciferase Assay

SH-SY5Y cells were seeded on 24-well plates and transfected in triplicates with 200 ng of wt Wnt3-GFP or Wnt3S212A-GFP or 75 ng of membrane-bound GFP as control together with 20 ng of firefly luciferase reporter pGL3 BAR (Biechele and Moon, 2008) and 5 ng of renilla luciferase reporter pGL4.73 hRLuc/SV40 (Promega, Madison, WI, USA) using Fugene HD Transfection Reagent (1 μg/1 μL, Promega). Twenty-four hours after transfection, reporter activity was measured using the dual luciferase reporter assay kit (Promega, Madison, WI, USA). Statistical analysis was performed using Student's *t*-test. Error bars represent SD, where ^***^p < 0.001, ^**^p < 0.01, and ^*^p < 0.05. HEK293T cells were transfected with wt Wnt3-EGFP or S212A Wnt3-EGFP by electroporation using the Neon® Transfection System (Invitrogen, Singapore) according to the manufacturer's protocol. The transfected cells were incubated for ~24 h, washed with Hank's Balanced Salt Solution (HBSS; Invitrogen, Singapore) twice, and imaged using imaging medium (Phenol red free DMEM + 10% FBS). For cholesterol extraction measurements, 3 mM methyl-β-cyclodextrin (MßCD; Sigma-Aldrich) dissolved in HBSS was added to cells and incubated for 15 min. The treated cells were then washed with HBSS twice and measured in imaging medium.

### DRM Flotation

Capped sense RNAs of wt Wnt3-GFP or Wnt3S212A-GFP injected into one-cell zebrafish embryos. Embryos were processed for detergent-resistant membrane (DRM) flotation protocol at 7 hpf. Embryos were collected in 1.5 mL test tubes, washed with ice-cold 1X TNE buffer (50 mM Tris [pH 7.4], 150 mM NaCl, and 2 mM EDTA). Embryos were lysed in 400 μL 1X TNE buffer containing phosphatase inhibitor (PI; Roche, Basel, Switzerland), protease inhibitor cocktail (PIC; Sigma-Aldrich, St. Louis, MO, USA) and 1% Triton X-100. Samples were then passed 20 times through a 26G needle, incubated on ice for 30 min and centrifuged at 4°C at 3,000 g for 5 min. Four hundred microliters of supernatant was homogenously mixed with 800 μL of OptiPrep Density Gradient Medium (60%, Sigma-Aldrich). Gradients were prepared by overlaying this mixture gently with 1,680 μL of 30% OptiPrep in 1X TNE and 720 μL of 5% Optiprep in 1X TNE (Sezgin et al., 2017a). Samples were ultracentrifuged at 226,800 g (45,000 rpm) for 6 h at 4°C using a type 90 Ti rotor in an Optima L-100 XP ultracentrifuge. Eight fractions were collected as 400 μL each. Proteins were precipitated by mixing the samples with 10% TCA and incubating for 15 min at −20°C. Samples were then centrifuged at 17,000 g at 4°C for 1 h. After centrifugation, supernatant was discarded, pellet was dissolved in sample loading dye and western blotting was performed.

### Co-immunoprecipitation (CoIP)

HEK293T cells were seeded in 6-well plates. Cells were transfected with Zf wt Wnt3-GFP or ZfWnt3S212A-GFP together with Zf FLAG-Fz8a. Forty-eight hours after transfection, cells were washed with ice-cold PBS and lysed with NOP buffer [10 mM Hepes KOH pH 7.4, 150 mM NaCl, 2 mM EDTA, 10% glycerol, 1% NP40 (Igepal CA-630, Sigma-Aldrich)]. Samples were passed 20 times through a 21G needle and centrifuged at 300 g for 5 min at 4°C. Supernatant was precipitated with Dynabeads using Dynabeads Protein G Co-Immunoprecipitation Kit (Thermo Fisher Scientific) according to the kit protocol.

### Secretion Assay

HEK293T cells were seeded in 10-cm culture plates. Cells were transfected with Zf wt Wnt3-GFP or ZfWnt3S212A-GF. Membrane-bound GFP and secreted GFP and were used as negative and positive controls, respectively. The day cells reached 100% confluence was accepted as day 0. Media was collected at day 2, 4, and 6, filtered with Minisart filters with a pore size of 0.2 μm (Merck, Burlington, MA, USA) and media was concentrated with Amicon Ultra-4 Centrifugal Filter Unit (Merck, Burlington, MA, USA). The viability and continuous growth of the collected cells was verified by MTT [3-(4,5-Dimethylthiazol-2-yl)-2,5-Diphenyltetrazolium Bromide] assay (Figure S5). For MTT assay, cells were seeded in a 96-well plate (5,000 cells/100 μL). 15 microliters of MTT reagent (5 mg/ml) was added onto the cells at 80% confluence (day-1), day 0, 2, 4, and 6. After 4 h incubation, media were removed and 100 μL of DMSO was added into each well. Cells were incubated for 30 min in dark. Absorbances were recorded at 570 nm. Samples were immunoprecipitated with an anti-GFP (see below) antibody using Dynabeads™ Protein G, prepared with 5X loading dye and processed for western blotting.

### Western Blotting

Samples were dissolved in 5X loading dye and separated by SDS gel electrophoresis by running on 10% acrylamide-bisacrylamide gel. Proteins were transferred to polyvinylidene fluoride (PVDF) membrane (GE Healthcare Life science, Chicago, IL, USA). Membrane blots was blocked in 5% milk powder for 45 min at room temperature (RT). Following antibodies were used at the indicated dilutions for membrane incubation. Primary antibodies: rabbit anti-GFP [(D5.1) XP, 1: 1000; Cell Signaling Technology, Danvers, MA, USA] rabbit anti-TfR2 (1:2000; Abcam, Cambridge, UK), mouse anti-Caveolin1 (1:2000; BD Transduction Laboratories, Franklin Lakes, NJ, USA) and anti-DDDDK tag antibody (ab1162, Abcam). Secondary antibodies: rabbit IgG HRP-linked F(ab′)2 fragment from donkey (1: 2500; GE Healthcare Bio-Sciences) and mouse IgG HRP Linked F(ab′)2 fragment from sheep (1:2500; GE Healthcare Bio-Sciences).

### ITIR-FCS Measurements and Imaging FCS Diffusion Law

To investigate the membrane organization and dynamics of Wnt3 and its acylation site mutant, Imaging Total Internal Reflection Fluorescence Correlation Spectroscopy (ITIR-FCS) was performed on HEK293T cells transfected with wt Wnt3-EGFP or S212A Wnt3-EGFP. The transfected cells were mounted on an Olympus Inverted epi-fluorescence microscope IX83 with motorized TIRF illumination combiner (cell^∧^TIRF/IX3-MITICO, Olympus). The cells were illuminated with 100 μW of a 488 nm laser (Olympus Cell Lasers), which was reflected to the back focal plane of an Olympus UApoN 100x/1.49 oil immersion objective using a ZT 405/488/561/640rpc (Chroma Technology, USA) dichroic mirror. The fluorescence signal was collected by the same objective and then was filtered using a quad band ZET 405/488/561/647m emission filter for TIRF applications (Chroma Technology, USA), and directed to an Andor iXon3 X-9388 EMCCD camera (128 × 128 pixels, 24 μm pixel size). All measurements were performed at 37°C and 5% CO_2_ by fitting an incubator with an objective heater (Live Cell Instrument, CU-109, Chamlide, Seoul, Korea) and a CO_2_/Air gas chamber (Live Cell Instrument, FC-5, Chamlide, Seoul, Korea) to the stage of the TIRF microscope. TIRF mode was achieved by adjusting the incident angle of illumination using the Olympus Xcellence software, thereby illuminating only the cell membrane. For data acquisition, a 21 × 21 pixel region was chosen in the center of the cell (sample plane of 5 × 5 μm^2^), and 50,000 frames were acquired at 3 ms exposure. The obtained stacks were analyzed using the ImFCS plugin (http://www.dbs.nus.edu.sg/lab/BFL/imfcs_image_j_plugin.html) for Image J. Analysis of the intensity fluctuations was achieved via autocorrelation functions (ACF curves), which were fitted using equation (1) to obtain diffusion coefficient and number of particles maps. The ImFCS diffusion law (τ_D_ vs. *A*_*eff*_) was plotted to obtain the τ_0_ values which gives information about membrane organization and dynamics of the probe.

(1) $$\begin{array}{l} G\left(\tau \right)=\;\sum_{i}^{N_{d}}\alpha_{i}{\left[\frac{\mathrm{erf}\left(p\left(\tau \right)\right)+\;\frac{\left(\exp-{\left(p\left(\tau \right)\right)}^{2}-1\right)}{\sqrt{\pi \;}p\left(\tau \right)}}{\mathrm{erf}\left(p\left(0\right)\right)+\;\frac{\left(\text{exp-}{\left(\text{p}\left(\text{0}\right)\right)}^{\text{2}}\text{-1}\right)}{\sqrt{\pi }\;p\left(0\right)}}\right]}^{2}\\ \;\left[1+\left(\frac{F_{t}}{1-F_{t}}\right)\exp\left(-\frac{\tau }{t_{f}}\right)\right]+G_{\infty }; \end{array}$$

Where $p\left(\tau \right)=\;\frac{a}{\sqrt{4D\tau +\omega_{0}^{2}}}$ and $\alpha_{i}=\frac{{\;B}_{i}^{2}\langle N_{i}\rangle }{\underset{i\;}{\sum }{\left(B_{i}\langle N_{i}\rangle \;\right)}^{2}}$

Here *G(*τ*)* is the temporal autocorrelation function containing *N*_*d*_ diffusive components, 〈*N*_*i*_〉 represents the average number of particles in the detection area, *B*_*i*_ represents the brightness of component i, *F*_*t*_ is the fraction of particles in triplet state, *a* is the pixel size, *t*_*f*_ is the average time molecules spend in the triplet state, G_∞_ is the convergence of G (τ) at long lag times and ω_0_ is the 1/e^2^ radius of the Gaussian approximation of the microscope's point spread function (PSF). The fitting parameters are *N, D, F*_*t*_*, t*_*f*_, and *G*_∞_.

The FCS diffusion law provides information on sub-resolution structures of membrane organization by measuring the dependence of the diffusion time of membrane probes at various observation areas (Wawrezinieck et al., 2005). The FCS diffusion law plot is generated by plotting the diffusion time (τ_D_) of a probe vs. the transverse area of the confocal volume. Freely diffusing particles exhibit a linear relationship between τ_D_ and the observation area, while particles undergoing hindered diffusion due to confinement in membrane domains or particles undergoing hop diffusion due to meshwork compartmentalization in the cell membranes display non-linear transitions in the FCS diffusion law plots (Sezgin et al., 2017a). Positive, negative and zero intercept (τ_0_) values indicate hindered diffusion with domain confinement, meshwork compartmentalized hop diffusion and free diffusion, respectively.

The FCS diffusion law can be implemented in imaging FCS (ImFCS), a camera based modality of FCS (Kannan et al., 2006; Sankaran et al., 2013), on either a Total Internal Reflection Fluorescence Microscope (TIRF) (Kannan et al., 2007) or a Single Plane Illumination Microscope (SPIM) (Wohland et al., 2010; Singh et al., 2013). In the ImFCS diffusion law, only a single measurement is required since various observation areas (*A*_*eff*_) can be achieved by software binning of pixels post acquisition, and convoluting the detection area with the PSF (Bag et al., 2016; Veerapathiran and Wohland, 2018). So, for a of pixel side length *a*, the observation area is *A*_*eff*_ = a^2^ ⊗ PSF. The ACFs for each observation area was calculated and fitted to obtain the diffusion time (τ_D_) in a given observation area. The ImFCS diffusion law was then plotted and fitted with the Equation (2) to obtain the τ_0_ value, and inverse slope of the plot would yield the effective diffusion coefficient (*D*_*eff*_).

(2) $$\begin{array}{r} \tau_{D}\left(A_{eff}\right)=\tau_{0}+\frac{A_{eff}}{D_{eff}} \end{array}$$

### Molecular Dynamics: Potential of Mean Force Calculations

Bilayers were constructed using insane (the MARTINI tool available at: cgmartini.nl/images/tools/insane/insane.py). Lipid parameters used were from Martini 2.1. The pulling simulations were performed using GROMACS version 4.6 (www.gromacs.org), with the MARTINI forcefield (Monticelli et al., 2008; de Jong et al., 2013), a pulling rate of 10 nm/ns and a pulling force constant of 10 kJ/mol/nm^2^. Starting positions for umbrella-sampling simulations were extracted from the pulling simulations spaced at distances of 0.1 nm (or 0.05 nm for the 10 lowest distance windows) along the reaction co-ordinate. Each umbrella sampling simulation was run for 1 μs, with a harmonic restraint of 1,000 kJ/mol/nm^2^ applied between the center of mass of lipid phosphate moieties and the lipidated Ser187 center of mass. The potential of mean force was extracted from the umbrella sampling simulations using the Weighted Histogram Analysis Method (WHAM) provided by the GROMACS g_wham tool (Hub et al., 2013). Periodic boundary conditions were applied, and a time step of 20 fs was used in all simulations. The temperature was maintained at 323 K using a Berendsen thermostat (Berendsen et al., 1984), and the pressure at 1 bar using a Berendsen barostat, and compressibility 5 ×10^−6^ bar. For both the temperature and pressure, a coupling constant of 4 ps was used for all simulations. In all simulations, the reaction field coulomb type was used with a switching function from 0.0 to 1.2 nm, and the Lennard Jones interactions were cutoff at 1.2 nm with a switching function applied from 0.9 nm. The LINCS algorithm was used to constrain covalent bonds to their equilibrium values (Hess et al., 1997).

## Results

### PAM and PLM Can Adopt a Diverse Range of Conformations

The Wnt proteins constitute a large family of secreted signaling molecules that are highly conserved among vertebrates. They are, however, less conserved between vertebrates and invertebrates while still exhibiting apparent orthologous relationships (Miller, 2002; Willert and Nusse, 2012) (Figure 1A). Another shared feature of Wnt proteins is the presence of a conserved serine amino acid that is palmitoylated (Figure 1A). Earlier, this palmitoylation was demonstrated to project from the conserved serine, located at the tip of Wnt's thumb into an extended groove within Fz8-CRD (Janda et al., 2012). As previous studies produced an ambiguity on the type of Wnt acylation due to experimental limitations (Takada et al., 2006; Janda et al., 2012), we initially set out to reveal whether the zebrafish canonical Wnt3 ligand is modified with a monounsaturated PAM or a saturated PLM at its conserved serine residue. For this purpose, we extracted PAM and PLM ligands from the PDB and aligned them on the resolved fatty acid structure of xWnt8-mouse Fz8-CRD complex (PDB id: 4F0A). This conformational analysis revealed that these both PLM and PAM flex into a diverse range of conformations (Figures 1B,C). It also highlighted that, unlike stated in the literature (Takada et al., 2006; Nile et al., 2017; Lee et al., 2019), both PAM and PLM could fold into a kinked conformation. Strikingly, among all published fatty acid coordinates, PLM has the highest probability to be the closest to 4F0A's fatty acid conformation (Figure 1D). Taken together with the ambiguities in determining the fatty acid type bound to the mouse Fz8 (Janda et al., 2012), we propose that 4F0A's fatty acid could very well be a PLM. This is also supported by the unrestrictive electron density of 4F0A's fatty acid ligand (Figure S1) (Janda et al., 2012). We also carried out potential of mean force calculations of PAM and PLM with pure membranes to predict their binding profile differences. Here, we simulated PAM and PLM binding to two different membranes with saturated or unsaturated lipids. These simulations did not reflect any difference between PAM or PLM membrane binding (Figure S2), suggesting that the fatty acid type is not a defining factor in the molecule's direct contact with the plasma membrane.

### PLM Modified Wnt Permits Conformationally Viable Fz8-PLM Interaction

**Figure 2:**
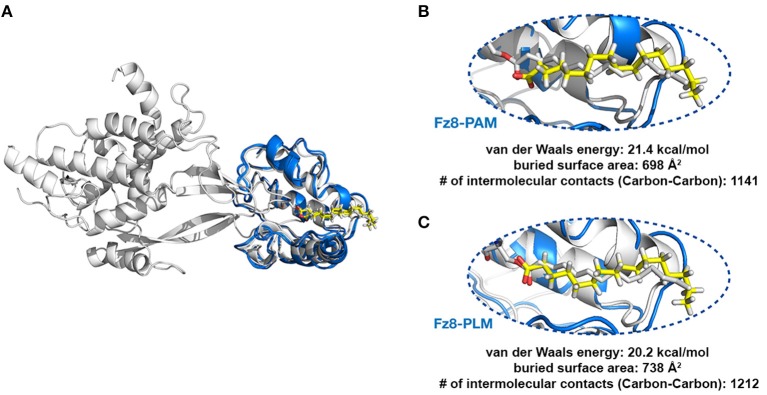
PLM modified Wnt permits conformationally viable Fz8-PLM interaction. **(A)** xWnt8-Fz8-PAM complex is depicted in white. xWnt8-Fz8 are represented in cartoon, where PAM is denoted in sticks. Modeled PAM and PLM ligands (including the previously missing atoms we added) are represented in yellow sticks. **(B)** A close-up into the Fz8-PAM interactions. Modeled PAM cannot be aligned on the oxygen atoms of 4F0A's fatty acid. **(C)** PLM can direct its oxygen atoms without any problems to the serine that is modified.

Next, we tested if PAM/PLM affects the binding of Wnt to Fz8. To this end, we designed two modeling scenarios, probing the structural differences between mouse Fz8-PAM and mouse Fz8-PLM interactions. For this, we constructed the atomistic models of mouse Fz8-PAM and mouse Fz8-PLM by using 4F0A's Fz8-fatty acid binding mode as a template (see section Materials and Methods, Figure 2A). Our Fz8-PAM and Fz8-PLM models unravel the following findings: (1) Both ligands are competent to reside along Fz8's hydrophobic channel. (2) However, only in the case of PLM the oxygen atoms of the fatty acid are close enough to be covalently linked to Ser187 of xWnt8 (compare Figures 2B,C and Figure S3). (3) Moreover, PLM induces a 6% increase in the buried surface area across the Fz8-fatty acid surface. Expanding on these observations, we generated the first structural model of zebrafish Fz8-PLM complex (see Materials and Methods) and PLM turned out to be compatible with zebrafish Fz8, similar to mouse Fz8. The critical residues of PLM interacting with the zebrafish Fz8 are highlighted in Figure S4. These results indicate that even though free PAM/PLM can interact with Fz8 in a similar manner, only Fz8-PLM is conformationally competent with Wnt's acylation.

### Acylation of Wnt3 at the Conserved Serine Residue (S212) Is Not Necessary for Its Secretion and Interaction With Its Receptor

**Figure 3:**
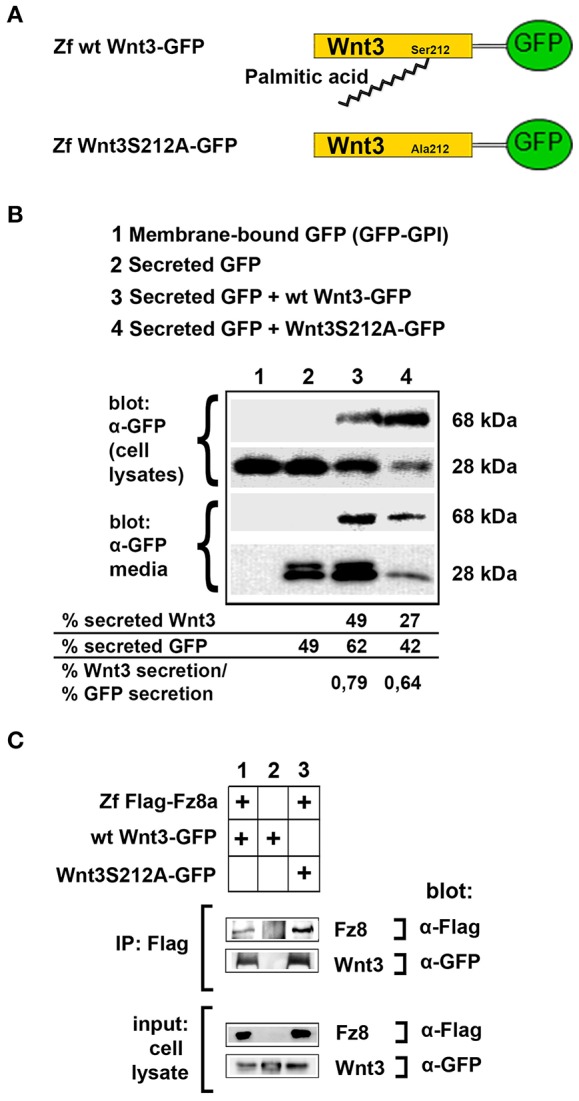
Acylation of Wnt3 at the conserved serine residue (S212) is not necessary for its secretion and interaction with its receptor. **(A)** Domain structure of C-terminally GFP-tagged wild-type (Zf wt Wnt3-GFP) and mutant (zf Wnt3S121A-GFP) zebrafish Wnt3. Palmitic acid is attached to the conserved serine residue. Palmitoylation is supposed not to occur in the mutant construct where serine is replaced by alanine. **(B)** Secretion assay for wt and mutant Wnt3 proteins and western blot of input (cell lysates) and collected media. Both wt Wnt3-GFP and Wnt3S121A-GFP are detected in the media collected from HEK293T cells transfected with corresponding constructs. Secreted and total produced levels of the proteins were calculated and used for comparisons. Membrane-bound GFP (GFP:GPI) and secreted GFP are used as negative (non-secretory protein) and positive (secretory protein) controls, respectively. Forty-nine, sixty-two, and forty-two percent of total GFP were secreted from cells transfected with secreted (sec) GFP, sec GFP+ wt Wnt3-GFP and sec GFP+ Wnt3S212A-GFP, respectively. Forty-nine and twenty-seven percent of Wnt3 were secreted from cells transfected with sec GFP+ wt Wnt3-GFP and sec GFP+ Wnt3S212A-GFP, respectively. Ratios of secreted Wnt3 to secreted GFP were 0.79 and 0.64 for wt Wnt and Wnt3S212A, respectively. Percentages represent mean of three independent experiments. **(C)** Coimmunoprecipitation of wt and mutant Wnt3 proteins with Frizzled8 receptor. Both wt Wnt3-GFP and Wnt3S121A-GFP coIP with Zf FLAG-Fz8a in HEK293T cells. wt Wnt3-GFP does not bind to Dynabeads non-specifically. Three independent experiments were performed.

To test the influence of acylation on canonical Wnt function, we first aimed to determine whether the lack of acylation alters Wnt secretion and interaction with its receptor. To this purpose, we cloned the wild-type zebrafish Wnt3 into a pCS2P+ expression vector that has EGFP, termed as wt Wnt3-GFP (Figure 3A, top). By introducing a point mutation in zebrafish Wnt3 gene corresponding to the conserved serine residue at position 212 (S212), we generated a mutant Wnt3 which we termed as Wnt3S212A-GFP and has its serine replaced by alanine (Figure 3A, bottom). Then, to test whether lack of this acylation on Wnt3 has an influence on its secretion, we transfected HEK293T cells with wt Wnt3-GFP or Wnt3S212A-GFP with membrane-bound GFP (GFP:GPI) as negative and secreted GFP as positive control alongside. When we compared the secreted and total produced levels of the proteins, both wt Wnt3 and mutant Wnt3, albeit reduced, were found to be secreted into the media (Figure 3B). This reduction in secretion level could also be due to the lower growth rate of cells transfected with the mutant Wnt3 (Figure S5). Secreted GFP used as control was detected in the media, whereas membrane-bound GFP control was not (Figure 3B). Finally, to understand how S212 acylation of Wnt3 affects its physical interaction to Fz8 receptor, we co-expressed wt Wnt3-GFP or Wnt3S212A-GFP with Zf FLAG-Fz8a. Both wt and mutant Wnt3, which exclusively represent the exogenously expressed proteins, coimmunoprecipitated (coIPed) with the exogenously expressed receptor Fz8a (Figure 3C). These collected data suggest that acylation of zebrafish Wnt3 at the conserved serine residue is dispensable for its secretion and binding to its receptor Fz8 at the plasma membrane.

### Acylation of Wnt3 Facilitates Its Partitioning Into More Ordered Plasma Membrane Environments

**Figure 4:**
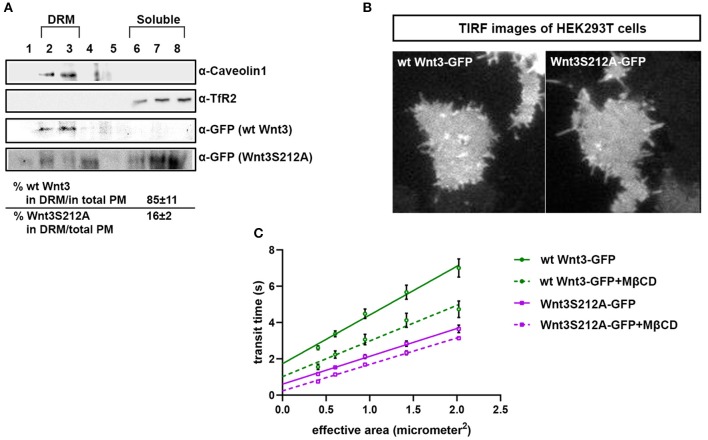
Acylation of Wnt3 facilitates its partitioning into more ordered plasma membrane environments. **(A)** Membrane flotation assay for wt and mutant Wnt constructs. Western blots show soluble and detergent-resistant fractions of plasma membranes derived from zebrafish embryos injected with 1 ng capped sense RNA of wt *Wnt3-GFP* or *Wnt3S212A-GFP*. DRMs and soluble fractions are marked by endogenous TfR2 and Caveolin-1 (Cav1), respectively. All Wnt proteins are detected by their GFP tags. Eighty-five percent of wt Wnt3-GFP is detected in the DRM fractions while only sixteen percent of mutant Wnt3S212A-GFP is detected in DRMs with the rest of it shifting to soluble phases. Percentages represent mean ± standard deviation (SD) of three independent experiments. **(B)** TIRF images of HEK293T cells expressing wt Wnt3-GFP and Wnt3S212A-GFP **(C)** ITIR-FCS measurements. Signal is recorded from the basolateral membrane of the cells and correlated with different bin size to form varying observation areas. The diffusion time positively correlates with the size of the area and determines the diffusion mode of the molecule as free or hindered. wt Wnt3-GFP undergoes domain-like diffusion (positive y-axis intercept) while Wnt3S212A-GFP tends to diffuse freely after cholesterol extraction. MßCD, methyl-β-cyclodextrin. Transit time and SD are obtained from three independent experiments.

We have previously shown that the canonical Wnt ligand preferably interacts with its receptors residing in the ordered membrane environments and undergoes cholesterol and saturated lipid-dependent domain-like diffusion at the plasma membrane (Sezgin et al., 2017a). To examine the impact of S212 acylation on Wnt binding to its receptor complex, we performed subcellular fractionation and subsequent membrane flotation assay on zebrafish embryos injected with the capped sense RNAs of wt *Wnt3-GFP* or *Wnt3S212A-GFP*. We found that wt *Wnt3* was enriched in the detergent resistant membrane (DRM) fractions marked by DRM marker Caveolin1. Though not being definitive, DRM enrichment shows a propensity of the molecules to partition into the ordered domains of the plasma membrane (Brown and London, 1998; Magee and Parmryd, 2003; Lingwood and Simons, 2007) (Figure 4A, compare third row to the first row). *Wnt3S212A-GFP*, however, significantly shifted from the DRMs toward the detergent soluble phases of the membrane marked by the transmembrane receptor Transferrin-receptor 2 (Tfr2) (Figure 4A, compare fourth row to the second row. Eighty-five percent of wt Wnt3 detected in DRMs and only 16% of Wnt3S212A detected in DRMs). To substantiate our findings obtained via *in vitro* DRM analysis with a less harsh, more sensitive and more accurate approach, we employed the Imaging Total Internal Reflection Fluorescence Correlation Spectroscopy (ITIR-FCS) diffusion law. In ITIR-FCS, due to internal reflection, an evanescent wave of light illuminates only the cell membrane and, unlike confocal-based FCS, the intracellular signals are not detected. Moreover, since it is a camera-based method, the fast diffusion that originates from the intracellular signal cannot be recorded, resulting in recording of signals coming almost exclusively from the plasma membrane. The diffusion behavior of molecules on the membrane can be investigated by analyzing the τ_0_ intercept values of the ImFCS diffusion law plots : a near zero intercept (τ_0_ ≤ ±0.2 s) indicates free diffusion, a positive intercept (τ_0_ > + 0.2 s) indicates domain confined diffusion due to membrane domains and a negative intercept (τ_0_ < −0.2 s) indicates hop-diffusion due to cytoskeleton compartmentalization (See Materials and Methods section). We expressed wt Wnt3-GFP or Wnt3S212A-GFP in HEK293T cells and evaluated the τ_0_ value (Figure 4B). Our analyses revealed that wt Wnt3-EGFP undergoes domain-like diffusion (τ_0_-value of 1.66 s ± 0.28 s) whereas Wnt3S212A-GFP shows reduced domain confinement with a lower y-axis intercept (τ_0_-value of 0.71 s ± 0.25 s). On extraction of cholesterol using 3 mM MßCD, τ_0_ converges to free diffusion at the plasma membrane for the mutant (τ_0_-value of 0.16 s ± 0.1 s), indicating the importance of acylation of Wnt3 at S212 for its association to membrane domains on the membrane (Figure 4C and Table 1). These data collectively show that acylation of Wnt3 is necessary for its proper binding in the ordered environments and its domain-confined diffusion at the plasma membrane.

**Table 1:** Y-axis intercept (τ_0_ values) for the ImFCS diffusion law of wt Wnt3 and Wnt3S212A mutant in HEK293 cells.

	**Untreated**	**3 mM MßCD**
wt-Wnt3	1.66 ± 0.28 s	0.78 ± 0.19 s
Wnt3S212A	0.71 ± 0.25 s	0.16 ± 0.1 s

### Acylation of Wnt3 Is Essential for Activation of Canonical Wnt Signaling

**Figure 5:**
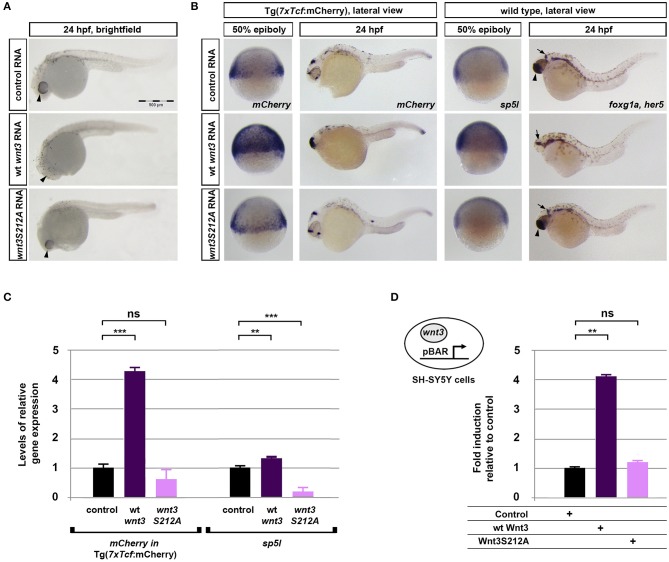
Acylation of Wnt3 is essential for activation of canonical Wnt signaling. **(A)** Morphological phenotypes at 24 hpf of embryos injected with capped sense RNA of *Wnt3-EGFP* (200 pg, 53/56 embryos) or *Wnt3(S212A)-EGFP* (200 pg, 46/49 embryos). **(B)** Whole mount *in situ* hybridization (WMISH) showing the loss of capacity in *Wnt3(S212A)-EGFP* (200 pg) capped sense RNA to activate canonical Wnt signaling. First and second columns left: *mCherry* WMISH shows upregulation of signaling in the transgenic Tg(*7xTcf-Xla.Siam:nlsm-Cherry*^*ia*^) canonical Wnt/ß-catenin reporter embryos by *Wnt3-EGFP* (36/36) but not by *Wnt3(S212A)-EGFP* (32/33). Second column right: Direct Wnt/ß-catenin target gene *sp5l* is upregulated by *Wnt3-EGFP* (30/32) but not by *Wnt3(S212A)-EGFP* (29/32). Rightmost column: *Wnt3-EGFP* (33/34), but not *Wnt3(S212A)-EGFP* (36/36), completely abolishes the telencephalon, marked by *foxg1a* (arrowhead), while the midbrain-hindbrain boundary, marked by *her5* (arrow), is preserved. **(C)** Expression levels of *mCherry* in transgenic Tg(7xTcf-Xla.Siam:nlsm-Cherry^ia^) and *sp5l* in wt embryos injected with capped sense RNAs of *Wnt3-EGFP* (200 pg) or *Wnt3(S212A)-EGFP* (200 pg) and determined by qPCR. Four independent experiments were performed. **(D)** Average and SD of the mean (error bars) values of pBAR luciferase reporter activity monitoring Wnt/ß-catenin signaling activity (normalized to renilla luciferase activity) in SH-SY5Y cells transfected with wt Wnt3-GFP or Wnt3S212A-GFP. wt Wnt3-GFP significantly activated the reporter whereas Wnt3S212A-GFP did not. Statistical significance was evaluated using unpaired *t*-test. ***p < 0.001, **p < 0.01, and n.s., non-significant. Error bars represent SD. Three independent experiments were performed.

To investigate whether acylation of Wnt3 at the conserved serine influences Wnt/β-catenin signaling activity, we injected the capped sense RNAs of wt *Wnt3-GFP* or *Wnt3S212A-GFP* into 1-cell zebrafish embryos. While wt *Wnt3-GFP* efficiently suppressed eye formation at 24 hpf, a distinctive phenotype caused by enhanced Wnt/ß-catenin signaling (Lekven et al., 2001; Ozhan et al., 2013), *Wnt3S212A-GFP* overexpression did not exhibit any phenotypic alteration as compared to control (Figure 5A). Next to directly test the specificity of this effect on Wnt/β-catenin signaling, we exploited a transgenic zebrafish reporter of Wnt/ß-catenin signaling Tg(*7xTcf-Xla.Siam*:nlsm-Cherry^ia^) (Moro et al., 2012). In contrast to strong activation of the reporter by wt *Wnt3-GFP* in embryos at both gastrula (50% epiboly) and organogenesis (24 hpf) stages, *Wnt3S212A-GFP* overexpression had no detectable effect on the reporter activity as shown by both WMISH at both stages and qPCR at the gastrula stage (Figure 5B, first and second columns, left; Figure 5C, left). *Wnt3S212A-GFP* appeared to reduce the expression of the direct Wnt/ß-catenin target gene *sp5l* (Weidinger et al., 2005) as detectable by WMISH and qPCR at the gastrula stage (Figure 5B, second column, right; Figure 5C, right). This reduction could be due to a potential dominant negative effect of the mutation. During vertebrate gastrulation, Wnt/β-catenin signaling is required for induction of posterior neural fates in neuroectodermal patterning (Lekven et al., 2001). wt *Wnt3-GFP* overexpression thus reduced the anterior neuroectodermal fates, as evidenced by complete elimination of the telencephalon marker *foxg1a* at organogenesis while the midbrain-hindbrain boundary marker *her5* is preserved (Figure 5B, rightmost column). On the contrary, *Wnt3S212A-GFP* expressing embryos appeared indifferent to the control (Figure 5B, first column, right). Next we tested the activity of the wt and mutant Wnt3 constructs in mammalian cells and found that Wnt3S212A-GFP, similarly to zebrafish embryos, was unable to activate on Wnt/β-catenin signaling in SH-SY5Y cells as evidenced by the activation of the pBAR reporter of Tcf/Lef-mediated transcription (Figure 5D). Thus, we conclude that acylation of Wnt3 at the conserved serine residue is required for Wnt3 to activate canonical Wnt signaling.

## Discussion

The dynamic lateral heterogeneity in the plasma membrane is known to control the formation of canonical Wnt-receptor complex by ensuring that the Wnt ligand preferentially binds to its receptors in ordered membrane environments where it activates downstream signaling pathway (Sezgin et al., 2017a). However, whether the posttranslational lipid modifications of Wnt influence this preferential binding remained to be understood. Our study uncovers the role of a specific lipid modification, acylation with a saturated fatty acid, of canonical Wnt ligand in regulation of its interactions with the receptor complex within the ordered membrane regions. Our computational, biochemical and biophysical data together suggest that (i) the canonical Wnt3 ligand is acylated at a conserved serine residue presumably with a saturated fatty acid PLM, (ii) this acylation, while being dispensable for its secretion and interaction with the Fz8 receptor, (iii) is essential for its proper partitioning into the ordered membrane domains, (iv) where Wnt can activate downstream canonical Wnt signaling. Therefore, the acylation appears to assist canonical Wnt in associating with the proper domains of the plasma membrane where it can activate the signaling.

Palmitoylation serves an essential function in protein activation through modifying hydrophobicity and conformation, and ultimately determining the protein's subcellular localization and function (Smotrys and Linder, 2004). Acylation of mWnt3a has been proposed to occur through addition of a palmitoleic acid, a monounsaturated fatty acid, to a conserved serine residue (Takada et al., 2006). This, however, does not properly align with (1) the preferential binding of the canonical Wnt ligand to its receptor complex in the ordered membrane domains (Sezgin et al., 2017a) and (2) the influence of steric preference for a saturated fatty acid on Wnt's domain confinement and signaling activity. In this work, the authors also speculated that the kinked nature of the unsaturated fatty acid, PAM is essential to its packing into the interior of small lipid particles (Takada et al., 2006). Other studies have likewise assumed that due to its unsaturated nature, only PAM exerts a kinked conformation, whereas PLM adopts a linear one (Nile et al., 2017; Lee et al., 2019). Lee et al. (2019) have further argued that the cavity of human Porcupine protein, a member of the membrane-bound O-acyl transferases catalyzing the posttranslational fatty acylation of Wnts, is likely to recognize the kink in the unsaturated fatty acyl substrate. Our thorough analysis of the available PAM and PLM conformers, however, has demonstrated that both ligands span a diverse range of conformations, from linear to kinked (Figure 1); excluding the possibility that binding of PAM or PLM is driven by shape selectivity. Thus, we suggest that Porcupine's cavity easily allows a saturated fatty acyl chain such as PLM to fit in by taking its shape.

While the structural basis of a lipid-modified Wnt recognition by Fz has been resolved, the full atomistic details of this lipid modification has remained unclear (Janda et al., 2012). The fact that Janda et al. could not unambiguously determine the chemical identity of the lipid bound to mouse Fz8 by mass spectrometry (Janda et al., 2012) induces an open discussion platform whether there is any inherent molecular specificity encoded in Fz8 toward PAM or PLM. In this work, we present the first structural evidence that mouse Fzd8 prefers to bind to PLM instead of PAM since only PLM could adopt a conformation compatible with the stereochemical features Wnt modification, while not entirely excluding the possibility that Wnt3 could also be modified by PAM. We believe that the saturation of this lipid facilitates binding of Wnt in the ordered domains of the plasma membrane where saturated lipids are enriched. A promising question would be to compare how differently Wnt ligands with saturated and unsaturated lipid modifications behave in binding to the membrane domains. PLM binding is also endorsed by the increased buried surface area, indicating a tighter interaction with Fz8. In addition, transferring these findings to an analogous zebrafish system reveals the zebrafish Fz8 amino acids that are potentially critical to its interaction with a PLM molecule. Thus, it will be very interesting to further test how these amino acids influence binding of Fz8 to the PLM of Wnt3.

Independent studies on mWnt1 and mWnt3a showed that secretion of lipid-deficient Wnts were significantly reduced and these mutants displayed significantly less signaling activity than wild-type Wnts (Takada et al., 2006; Galli and Burrus, 2011; Gao and Hannoush, 2014). In contrast, the same ligands without lipidic adducts at their conserved serine residues were found to be secreted normally and showed no significant differences in their abilities to bind the Wnt receptor Fz4 as compared to their wild-type counterparts (Doubravska et al., 2011). These acyl-deficient Wnts, however, were not able to activate signaling, implying that acylation is indispensable for proper activation of the canonical Wnt pathway (Doubravska et al., 2011). Similarly, *Drosophila* Wg mutant WgS239A, where acylation is prevented due to replacement of its conserved serine residue with an alanine, was shown to be secreted normally into the media of *Drosophila* S2 cells, but displayed poor signaling activity (Franch-Marro et al., 2008). A recent work has likewise reported that mutation of the conserved acylation site of xWnt8 or mWnt1 interferes neither with its secretion into the culture medium when expressed in HEK293 or Expi293 cells nor with its interaction with the Fz receptor (Speer et al., 2019). The ability of several non-acylated Wnts including xWnt8S187A, mWnt1S224A and hWnt3aS209A to activate signaling varied significantly and even contrasted for mWnt1S224A between *in vitro* and *in vivo*, arguing that, acylation dependence varies between different Wnt ligands and that this dependence can even be regulated in a biological context-dependent manner (Speer et al., 2019). Our results show that S212 acylation of zebrafish Wnt3 presumably with a saturated fatty acid, while reducing its secretion, is largely dispensable for its secretion and binding to its receptor Fz8 at the plasma membrane, but is essential for activation of signaling. Thus, we believe that, the role of acylation in protein secretion, receptor interaction and pathway activation varies between Wnt ligands, so it needs to be elucidated for each ligand individually and compared in different cellular contexts. Individual and context-dependent characterization of Wnt ligands will also yield important information on how to modulate Wnt signaling activity in different types of cancers. Moreover, Janda et al. reported that cysteine 55 (C55) residue of XWnt8 does not serve as a lipidation site but is rather engaged in a disulfide bond (Janda et al., 2012). Given the major role of lipidation in Wnt signaling, more work is required to elucidate whether indeed C80 in zebrafish Wnt3, the cysteine residue analogous to C55 in XWnt8 and C77 in mouse Wnt3a, is not lipidated.

Although Wnt/β-catenin signaling pathway have been extensively explored for the development of new drugs that inhibit canonical Wnt signaling, so far, no drugs have been approved to target the pathway (Zimmerman et al., 2012; Anastas and Moon, 2013; Bao et al., 2013; Kahn, 2014; Nusse and Clevers, 2017; Krishnamurthy and Kurzrock, 2018). A considerable amount of drugs that has entered the clinical trials consist of antibodies that target Fz CRDs at the plasma membrane (Gurney et al., 2012; Lee et al., 2015; Le et al., 2015; Tran and Zheng, 2017; Krishnamurthy and Kurzrock, 2018; Zeng et al., 2018). However, the fact that these antibodies target Fz receptors ubiquitously in a domain-independent manner contradicts the fact that the receptor pool, which can be activated by ligand binding, localizes in the ordered membrane domains (Sezgin et al., 2017a). In addition to domain-dependent activation of the receptor pool by the Wnt ligand, we show that acylation of Wnt is necessary for signaling, rendering domain-specific receptor targeting even more important for drug development strategies. Our study reveals that acylation of canonical Wnt ligand is critical for regulation of signaling activity via controlling the ligand's physical interactions with the receptor and the plasma membrane. Considering the major differences between the plasma membranes of healthy and cancer cells, novel therapies based on targeting the cancer cell membrane in a lipid-specific manner is very likely to constitute a promising approach for anticancer drug development. One such approach would be to modify candidate Fz CRD inhibitor molecules with proper lipid groups such as PLM to target them specifically to the Fz receptors residing in the ordered membrane domains and this way to convert them into more potent therapeutic agents to inhibit abnormal Wnt signaling.

## Data Availability Statement

The datasets generated for this study can be found in the https://github.com/ezgikaraca/Wnt-acylation.

## Ethics Statement

The animal study was reviewed and approved by Dokuz Eylul University-Izmir International Biomedicine and Genome Institute-Animal Experiments Local Ethics Committee.

## Author Contributions

GO, YA, ES, and EK designed the experiments. YA and GO performed the biochemical and developmental experiments (Figures 3, 4A, 5 and Figure S5). OO and EK conducted the structural modeling experiments (Figures 1, 2 and Figures S1, S3, S4, S6). SV performed the biophysical experiments (Figures 4B,C). AD performed the molecular dynamics experiments (Figure S2). MS, CE, and TW contributed reagents and materials analysis tools. GO, ES, and EK wrote the manuscript. All authors contributed to the discussion.

### Conflict of Interest

The authors declare that the research was conducted in the absence of any commercial or financial relationships that could be construed as a potential conflict of interest.

## References

[B1] Agarwal S. R. Gratwohl J. Cozad M. Yang P. C. Clancy C. E. Harvey R. D. (2018). Compartmentalized cAMP signaling associated with lipid raft and non-raft membrane domains in adult ventricular myocytes. Front. Pharmacol. 9:332. 10.3389/fphar.2018.00332 29740315 PMC5925456

[B2] Anastas J. N. Moon R. T. (2013). WNT signalling pathways as therapeutic targets in cancer. Nat. Rev. Cancer 13, 11–26. 10.1038/nrc3419 23258168

[B3] Angers S. Moon R. T. (2009). Proximal events in Wnt signal transduction. Nat. Rev. Mol. Cell Biol. 10, 468–477. 10.1038/nrm2717 19536106

[B4] Badawy S. M. M. Okada T. Kajimoto T. Hirase M. Matovelo S. A. Nakamura S. I.. (2018). Extracellular alpha-synuclein drives sphingosine 1-phosphate receptor subtype 1 out of lipid rafts, leading to impaired inhibitory G-protein signaling. J. Biol. Chem. 293, 8208–8216. 10.1074/jbc.RA118.001986 29632069 PMC5971450

[B5] Bag N. Ng X. W. Sankaran J. Wohland T. (2016). Spatiotemporal mapping of diffusion dynamics and organization in plasma membranes. Methods Appl. Fluoresc. 4:034003. 10.1088/2050-6120/4/3/034003 28355150

[B6] Bao J. Lee H. J. Zheng J. J. (2013). Genome-wide network analysis of Wnt signaling in three pediatric cancers. Sci. Rep. 3:2969. 10.1038/srep02969 24132329 PMC3797983

[B7] Berendsen H. J. C. Postma J. P. M. van Gunsteren W. F. Di Nola A. Haak J. R. (1984). Molecular dynamics with coupling to an external bath. J. Chem. Phys. 81, 3684–3690. 10.1063/1.448118

[B8] Biechele T. L. Moon R. T. (2008). Assaying beta-catenin/TCF transcription with beta-catenin/TCF transcription-based reporter constructs. Methods Mol. Biol. 468, 99–110. 10.1007/978-1-59745-249-6_8 19099249

[B9] Brown D. A. London E. (1998). Functions of lipid rafts in biological membranes. Annu. Rev. Cell Dev. Biol. 14, 111–136. 10.1146/annurev.cellbio.14.1.111 9891780

[B10] Clevers H. (2006). Wnt/beta-catenin signaling in development and disease. Cell 127, 469–480. 10.1016/j.cell.2006.10.018 17081971

[B11] Clevers H. Nusse R. (2012). Wnt/beta-catenin signaling and disease. Cell 149, 1192–1205. 10.1016/j.cell.2012.05.012 22682243

[B12] de Jong D. H. Singh G. Bennett W. F. Arnarez C. Wassenaar T. A. Schafer L. V.. (2013). Improved parameters for the martini coarse-grained protein force field. J. Chem. Theory Comput. 9, 687–697. 10.1021/ct300646g 26589065

[B13] Dinic J. Riehl A. Adler J. Parmryd I. (2015). The T cell receptor resides in ordered plasma membrane nanodomains that aggregate upon patching of the receptor. Sci. Rep. 5:10082. 10.1038/srep10082 25955440 PMC5386217

[B14] Doubravska L. Krausova M. Gradl D. Vojtechova M. Tumova L. Lukas J.. (2011). Fatty acid modification of Wnt1 and Wnt3a at serine is prerequisite for lipidation at cysteine and is essential for Wnt signalling. Cell Signal. 23, 837–848. 10.1016/j.cellsig.2011.01.007 21244856

[B15] Franch-Marro X. Wendler F. Griffith J. Maurice M. M. Vincent J. P. (2008). *In vivo* role of lipid adducts on Wingless. J. Cell Sci. 121, 1587–1592. 10.1242/jcs.015958 18430784 PMC7611555

[B16] Galli L. M. Burrus L. W. (2011). Differential palmit(e)oylation of Wnt1 on C93 and S224 residues has overlapping and distinct consequences. PLoS ONE 6:e26636. 10.1371/journal.pone.0026636 22046319 PMC3202554

[B17] Gao X. Hannoush R. N. (2014). Single-cell imaging of Wnt palmitoylation by the acyltransferase porcupine. Nat. Chem. Biol. 10, 61–68. 10.1038/nchembio.1392 24292069

[B18] Gurney A. Axelrod F. Bond C. J. Cain J. Chartier C. Donigan L.. (2012). Wnt pathway inhibition via the targeting of Frizzled receptors results in decreased growth and tumorigenicity of human tumors. Proc. Natl. Acad. Sci. U.S.A. 109, 11717–11722. 10.1073/pnas.1120068109 22753465 PMC3406803

[B19] Guven-Maiorov E. Keskin O. Gursoy A. VanWaes C. Chen Z. Tsai C. J.. (2015). The architecture of the TIR domain signalosome in the toll-like receptor-4 signaling pathway. Sci. Rep. 5:13128. 10.1038/srep13128 26293885 PMC4544004

[B20] Hess B. Bekker H. Berendsen H. J. C. Fraaije J. G. E. M. (1997). A linear constraint solver for molecular simulations. J. Comp. Chem. 18, 1463–1472.

[B21] Huang H. He X. (2008). Wnt/beta-catenin signaling: new (and old) players and new insights. Curr. Opin. Cell Biol. 20, 119–125. 10.1016/j.ceb.2008.01.009 18339531 PMC2390924

[B22] Hub J. S. de Groot B. L. van der Spoel D. (2013). g_wham—a free weighted histogram analysis implementation including robust error and autocorrelation estimates. J. Chem. Theory Comput. 6, 3713–3720. 10.1021/ct100494z

[B23] Janda C. Y. Waghray D. Levin A. M. Thomas C. Garcia K. C. (2012). Structural basis of Wnt recognition by Frizzled. Science 337, 59–64. 10.1126/science.1222879 22653731 PMC3577348

[B24] Jowett T. Lettice L. (1994). Whole-mount in situ hybridizations on zebrafish embryos using a mixture of digoxigenin- and fluorescein-labelled probes. Trends Genet. 10, 73–74. 10.1016/0168-9525(94)90220-8 8178366

[B25] Jury E. C. Flores-Borja F. Kabouridis P. S. (2007). Lipid rafts in T cell signalling and disease. Semin. Cell Dev. Biol. 18, 608–615. 10.1016/j.semcdb.2007.08.002 17890113 PMC2596300

[B26] Kahn M. (2014). Can we safely target the WNT pathway? Nat. Rev. Drug Discov. 13, 513–532. 10.1038/nrd4233 24981364 PMC4426976

[B27] Kannan B. Guo L. Sudhaharan T. Ahmed S. Maruyama I. Wohland T. (2007). Spatially resolved total internal reflection fluorescence correlation microscopy using an electron multiplying charge-coupled device camera. Anal. Chem. 79, 4463–4470. 10.1021/ac0624546 17489557

[B28] Kannan B. Har J. Y. Liu P. Maruyama I. Ding J. L. Wohland T. (2006). Electron multiplying charge-coupled device camera based fluorescence correlation spectroscopy. Anal. Chem. 78, 3444–3451. 10.1021/ac0600959 16689548

[B29] Kikuchi A. Yamamoto H. (2007). Regulation of Wnt signalling by receptor-mediated endocytosis. J. Biochem. 141, 443–451. 10.1093/jb/mvm061 17317692

[B30] Komekado H. Yamamoto H. Chiba T. Kikuchi A. (2007). Glycosylation and palmitoylation of Wnt-3a are coupled to produce an active form of Wnt-3a. Genes Cells 12, 521–534. 10.1111/j.1365-2443.2007.01068.x 17397399

[B31] Krishnamurthy N. Kurzrock R. (2018). Targeting the Wnt/beta-catenin pathway in cancer: update on effectors and inhibitors. Cancer Treat Rev. 62, 50–60. 10.1016/j.ctrv.2017.11.002 29169144 PMC5745276

[B32] Kurayoshi M. Yamamoto H. Izumi S. Kikuchi A. (2007). Post-translational palmitoylation and glycosylation of Wnt-5a are necessary for its signalling. Biochem J. 402, 515–523. 10.1042/BJ20061476 17117926 PMC1863570

[B33] Le P. N. McDermott J. D. Jimeno A. (2015). Targeting the Wnt pathway in human cancers: therapeutic targeting with a focus on OMP-54F28. Pharmacol. Ther. 146, 1–11. 10.1016/j.pharmthera.2014.08.005 25172549 PMC4304994

[B34] Lee C. J. Rana M. S. Bae C. Li Y. Banerjee A. (2019). In vitro reconstitution of Wnt acylation reveals structural determinants of substrate recognition by the acyltransferase human porcupine. J. Biol. Chem. 294, 231–245. 10.1074/jbc.RA118.005746 30420431 PMC6322882

[B35] Lee H. J. Bao J. Miller A. Zhang C. Wu J. Baday Y. C.. (2015). Structure-based discovery of novel small molecule wnt signaling inhibitors by targeting the cysteine-rich domain of frizzled. J. Biol. Chem. 290, 30596–30606. 10.1074/jbc.M115.673202 26504084 PMC4683279

[B36] Lekven A. C. Thorpe C. J. Waxman J. S. Moon R. T. (2001). Zebrafish wnt8 encodes two wnt8 proteins on a bicistronic transcript and is required for mesoderm and neurectoderm patterning. Dev. Cell 1, 103–114. 10.1016/S1534-5807(01)00007-7 11703928

[B37] Levental I. Lingwood D. Grzybek M. Coskun U. Simons K. (2010). Palmitoylation regulates raft affinity for the majority of integral raft proteins. Proc. Natl. Acad. Sci. U.S.A. 107, 22050–22054. 10.1073/pnas.1016184107 21131568 PMC3009825

[B38] Lingwood D. Simons K. (2007). Detergent resistance as a tool in membrane research. Nat. Protoc. 2, 2159–2165. 10.1038/nprot.2007.294 17853872

[B39] Logan C. Y. Nusse R. (2004). The Wnt signaling pathway in development and disease. Annu. Rev. Cell Dev. Biol. 20, 781–810. 10.1146/annurev.cellbio.20.010403.113126 15473860

[B40] Luz M. Spannl-Muller S. Ozhan G. Kagermeier-Schenk B. Rhinn M. Weidinger G.. (2014). Dynamic association with donor cell filopodia and lipid-modification are essential features of Wnt8a during patterning of the zebrafish neuroectoderm. PLoS ONE 9:e84922. 10.1371/journal.pone.0084922 24427298 PMC3888416

[B41] MacDonald B. T. Tamai K. He X. (2009). Wnt/beta-catenin signaling: components, mechanisms, and diseases. Dev. Cell 17, 9–26. 10.1016/j.devcel.2009.06.016 19619488 PMC2861485

[B42] Magee A. I. Parmryd I. (2003). Detergent-resistant membranes and the protein composition of lipid rafts. Genome Biol. 4:234. 10.1186/gb-2003-4-11-234 14611651 PMC329107

[B43] Martin A. C. R. Porter C. T. (2009). ProFit Version 3.1. London: University College London. Available online at: http://www.bioinf.org.uk/software/profit/index.html

[B44] Mason J. O. Kitajewski J. Varmus H. E. (1992). Mutational analysis of mouse Wnt-1 identifies two temperature-sensitive alleles and attributes of Wnt-1 protein essential for transformation of a mammary cell line. Mol. Biol. Cell 3, 521–533. 10.1091/mbc.3.5.521 1535241 PMC275605

[B45] Midgley A. C. Rogers M. Hallett M. B. Clayton A. Bowen T. Phillips A. O.. (2013). Transforming growth factor-beta1 (TGF-beta1)-stimulated fibroblast to myofibroblast differentiation is mediated by hyaluronan (HA)-facilitated epidermal growth factor receptor (EGFR) and CD44 co-localization in lipid rafts. J. Biol. Chem. 288, 14824–14838. 10.1074/jbc.M113.451336 23589287 PMC3663506

[B46] Miller J. R. (2002). The Wnts. Genome Biol. 3:1028. 10.1186/gb-2002-3-10-reviews1028 11806834 PMC150458

[B47] Monticelli L. Kandasamy S. K. Periole X. Larson R. G. Tieleman D. P. Marrink S. J. (2008). The MARTINI coarse-grained force field: extension to proteins. J. Chem. Theory Comput. 4, 819–834. 10.1021/ct700324x 26621095

[B48] Moro E. Ozhan-Kizil G. Mongera A. Beis D. Wierzbicki C. Young R. M.. (2012). *In vivo* Wnt signaling tracing through a transgenic biosensor fish reveals novel activity domains. Dev. Biol. 366, 327–340. 10.1016/j.ydbio.2012.03.023 22546689

[B49] Niehrs C. Shen J. (2010). Regulation of Lrp6 phosphorylation. Cell Mol. Life Sci. 67, 2551–2562. 10.1007/s00018-010-0329-3 20229235 PMC11115861

[B50] Nile A. H. Mukund S. Stanger K. Wang W. Hannoush R. N. (2017). Unsaturated fatty acyl recognition by Frizzled receptors mediates dimerization upon Wnt ligand binding. Proc. Natl. Acad. Sci. U.S.A. 114, 4147–4152. 10.1073/pnas.1618293114 28377511 PMC5402412

[B51] Nusse R. Clevers H. (2017). Wnt/beta-catenin signaling, disease, and emerging therapeutic modalities. Cell 169, 985–999. 10.1016/j.cell.2017.05.016 28575679

[B52] Ozhan G. Sezgin E. Wehner D. Pfister A. S. Kuhl S. J. Kagermeier-Schenk B.. (2013). Lypd6 enhances Wnt/beta-catenin signaling by promoting Lrp6 phosphorylation in raft plasma membrane domains. Dev. Cell 26, 331–345. 10.1016/j.devcel.2013.07.020 23987510

[B53] Ozhan G. Weidinger G. (2014). Restoring tissue homeostasis: Wnt signaling in tissue regeneration after acute injury, in Wnt Signaling in Development and Disease: Molecular Mechanisms and Biological Functions, eds Hoppler S. P. Moon R. T. (Hoboken, NJ: John Wiley & Sons, Inc). 10.1002/9781118444122.ch26

[B54] Sakane H. Yamamoto H. Kikuchi A. (2010). LRP6 is internalized by Dkk1 to suppress its phosphorylation in the lipid raft and is recycled for reuse. J. Cell Sci. 123, 360–368. 10.1242/jcs.058008 20053636

[B55] Sankaran J. Bag N. Kraut R. S. Wohland T. (2013). Accuracy and precision in camera-based fluorescence correlation spectroscopy measurements. Anal. Chem. 85, 3948–3954. 10.1021/ac303485t 23521662

[B56] Schuttelkopf A. W. van Aalten D. M. (2004). PRODRG: a tool for high-throughput crystallography of protein-ligand complexes. Acta Crystallogr. D Biol. Crystallogr. 60, 1355–1363. 10.1107/S0907444904011679 15272157

[B57] Sezgin E. Azbazdar Y. Ng X. W. Teh C. Simons K. Weidinger G.. (2017a). Binding of canonical Wnt ligands to their receptor complexes occurs in ordered plasma membrane environments. FEBS J. 284, 2513–2526. 10.1111/febs.14139 28626941 PMC5599997

[B58] Sezgin E. Levental I. Mayor S. Eggeling C. (2017b). The mystery of membrane organization: composition, regulation and roles of lipid rafts. Nat. Rev. Mol. Cell Biol. 18, 361–374. 10.1038/nrm.2017.16 28356571 PMC5500228

[B59] Simons K. Ikonen E. (1997). Functional rafts in cell membranes. Nature 387, 569–572. 10.1038/42408 9177342

[B60] Simons K. Toomre D. (2000). Lipid rafts and signal transduction. Nat. Rev. Mol. Cell Biol. 1, 31–39. 10.1038/35036052 11413487

[B61] Singh A. P. Krieger J. W. Buchholz J. Charbon E. Langowski J. Wohland T. (2013). The performance of 2D array detectors for light sheet based fluorescence correlation spectroscopy. Opt. Express 21, 8652–8668. 10.1364/OE.21.008652 23571955

[B62] Smotrys J. E. Linder M. E. (2004). Palmitoylation of intracellular signaling proteins: regulation and function. Annu. Rev. Biochem. 73, 559–587. 10.1146/annurev.biochem.73.011303.073954 15189153

[B63] Speer K. F. Sommer A. Tajer B. Mullins M. C. Klein P. S. Lemmon M. A. (2019). Non-acylated Wnts can promote signaling. Cell Rep. 26, 875–883. 10.1016/j.celrep.2018.12.104 30673610 PMC6429962

[B64] Takada R. Satomi Y. Kurata T. Ueno N. Norioka S. Kondoh H.. (2006). Monounsaturated fatty acid modification of Wnt protein: its role in Wnt secretion. Dev. Cell 11, 791–801. 10.1016/j.devcel.2006.10.003 17141155

[B65] Tang X. Wu Y. Belenkaya T. Y. Huang Q. Ray L. Qu J.. (2012). Roles of N-glycosylation and lipidation in Wg secretion and signaling. Dev. Biol. 364, 32–41. 10.1016/j.ydbio.2012.01.009 22285813 PMC3315154

[B66] The MathWorks (2018). MATLAB and Statistics Toolbox Release. Natick, MA.

[B67] Tran F. H. Zheng J. J. (2017). Modulating the wnt signaling pathway with small molecules. Protein Sci. 26, 650–661. 10.1002/pro.3122 28120389 PMC5368067

[B68] van Zundert G. C. P. Rodrigues J. Trellet M. Schmitz C. Kastritis P. L. Karaca E.. (2016). The HADDOCK2.2 web server: user-friendly integrative modeling of biomolecular complexes. J. Mol. Biol. 428, 720–725. 10.1016/j.jmb.2015.09.014 26410586

[B69] Veerapathiran S. Wohland T. (2018). The imaging FCS diffusion law in the presence of multiple diffusive modes. Methods 140–141, 140–150. 10.1016/j.ymeth.2017.11.016 29203404

[B70] Wawrezinieck L. Rigneault H. Marguet D. Lenne P. F. (2005). Fluorescence correlation spectroscopy diffusion laws to probe the submicron cell membrane organization. Biophys. J. 89, 4029–4042. 10.1529/biophysj.105.067959 16199500 PMC1366968

[B71] Weidinger G. Thorpe C. J. Wuennenberg-Stapleton K. Ngai J. Moon R. T. (2005). The Sp1-related transcription factors sp5 and sp5-like act downstream of Wnt/beta-catenin signaling in mesoderm and neuroectoderm patterning. Curr. Biol. 15, 489–500. 10.1016/j.cub.2005.01.041 15797017

[B72] Willert K. Brown J. D. Danenberg E. Duncan A. W. Weissman I. L. Reya T.. (2003). Wnt proteins are lipid-modified and can act as stem cell growth factors. Nature 423, 448–452. 10.1038/nature01611 12717451

[B73] Willert K. Nusse R. (2012). Wnt proteins. Cold Spring Harb. Perspect. Biol. 4:a007864. 10.1101/cshperspect.a007864 22952392 PMC3428774

[B74] Wohland T. Shi X. Sankaran J. Stelzer E. H. (2010). Single plane illumination fluorescence correlation spectroscopy (SPIM-FCS) probes inhomogeneous three-dimensional environments. Opt. Express 18, 10627–10641. 10.1364/OE.18.010627 20588915

[B75] Yamamoto H. Sakane H. Michiue T. Kikuchi A. (2008). Wnt3a and Dkk1 regulate distinct internalization pathways of LRP6 to tune the activation of beta-catenin signaling. Dev. Cell 15, 37–48. 10.1016/j.devcel.2008.04.015 18606139

[B76] Yang J. Yan R. Roy A. Xu D. Poisson J. Zhang Y. (2015). The I-TASSER Suite: protein structure and function prediction. Nat. Methods 12, 7–8. 10.1038/nmeth.3213 25549265 PMC4428668

[B77] Zeng C. M. Chen Z. Fu L. (2018). Frizzled receptors as potential therapeutic targets in human cancers. Int. J. Mol. Sci. 19:E1543. 10.3390/ijms19051543 29789460 PMC5983605

[B78] Zhai L. Chaturvedi D. Cumberledge S. (2004). Drosophila wnt-1 undergoes a hydrophobic modification and is targeted to lipid rafts, a process that requires porcupine. J. Biol. Chem. 279, 33220–33227. 10.1074/jbc.M403407200 15166250

[B79] Zimmerman Z. F. Moon R. T. Chien A. J. (2012). Targeting Wnt pathways in disease. Cold Spring Harb. Perspect. Biol. 4:a008086. 10.1101/cshperspect.a008086 23001988 PMC3536347

