# Supplementary material for: More Favorable Palmitic Acid Over Palmitoleic Acid Modification of Wnt3 Ensures Its Localization and Activity in Plasma Membrane Domains
Source: Front Cell Dev Biol. 2019 Nov 15;7:281. doi: 10.3389/fcell.2019.00281 (PMC6873803; doi:10.3389/fcell.2019.00281)
Supplement: Supplementary file 1 [file Table_1.docx]

**Supplementary Information for Azbazdar et. al.**

**Supplementary Figures and Figure Legends**


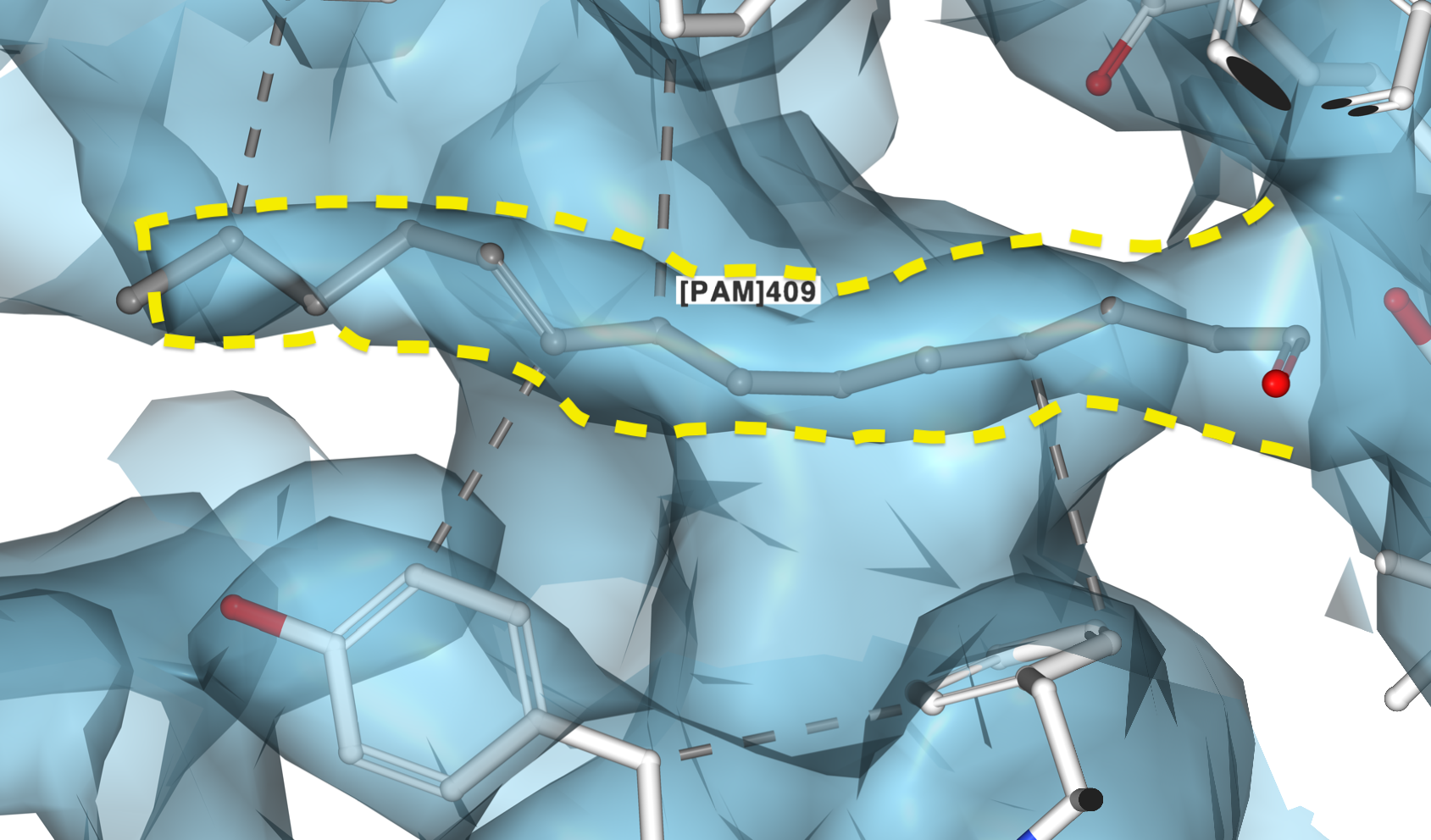


**Figure S1.** **Electron density defined around 4F0A’s PAM is not restrictive enough to unambiguously define its conformation.** The electron density of 4F0A’s PAM is encircled with yellow dashed lines. PAM is represented in dark gray sticks. The image is generated with the electron density viewing option of RSCB PDB (0.3 ISO level of 2fo-fc was used).


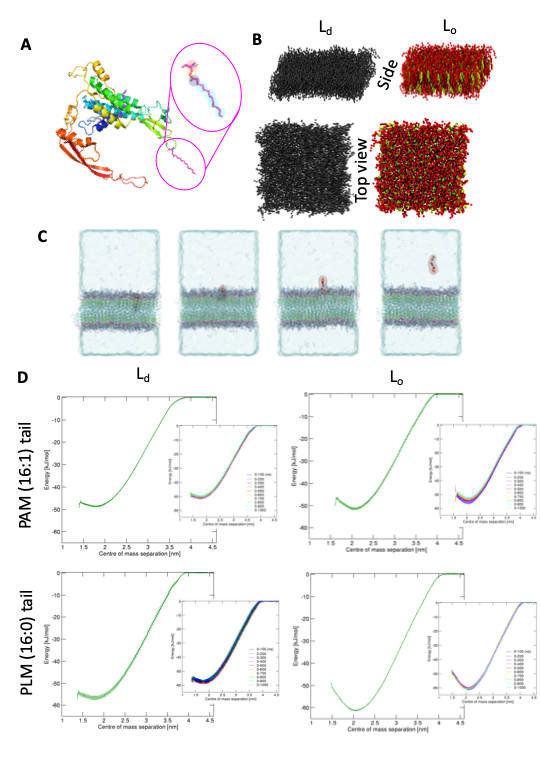


**Figure S2: Free energy of pulling lipidated Ser187 from ordered and disordered membranes.** (A): *Xenopus* Wnt8 (xWnt8) structure (Protein Data Bank [PDB] id: 4F0A) colored from blue (N-terminus) to red (C-terminus), with palmitoylated Ser187 shown as sticks in magenta. Inset shows an atomistic representation of Ser187 and palmitoylation, with coarse-grained MARTINI beads overlaid (pink bead, Ser187 backbone; yellow bead, Ser187 sidechain; dark blue bead, glycerol moiety; cyan beads, hydrocarbon tail moieties). (B) Side and top view of membranes that simulations were performed on. On the left, in grey, is a disordered POPC membrane; on the right is an ordered DPPC:CHOL 60:40 membrane, with DPPC in red, CHOL in yellow. Lipids are shown in their coarse-grained MARTINI representation. (C) Snapshots of the pulling simulation, with Ser187 plus palmitoyl group (outlined in red) being pulled from an ordered bilayer (lipids shown as beads) with the palmitoylated Ser187 embedded in the bilayer. (D) Potential of mean force calculations showing the free energy of pulling the lipidated Ser187 out of a disordered (left column) or ordered (right column) membrane, as a function of the separation from the center of mass of lipid molecules. Potential of mean force calculations were performed for Ser187 covalently bonded to: an unsaturated 16:1 PAM lipid tail (top row); and a saturated PLM lipid 16:0 tail (bottom row). The disordered membrane was modeled with 100 % POPC, while the ordered membrane was modeled with a 60:40 DPPC:CHOL ratio. Calculations were run on windows of 1µs, spaced at distances of 0.1 nm, or 0.05 nm for the 10 lowest distance windows. The graphs in the main panel use 0.5-1 µs from each simulation window. On each panel, the inset shows potential of mean force as calculated for the successive initial parts of the simulation windows, to demonstrate convergence. Error bars on all graphs were calculated by bootstrapping with 200 resamples.


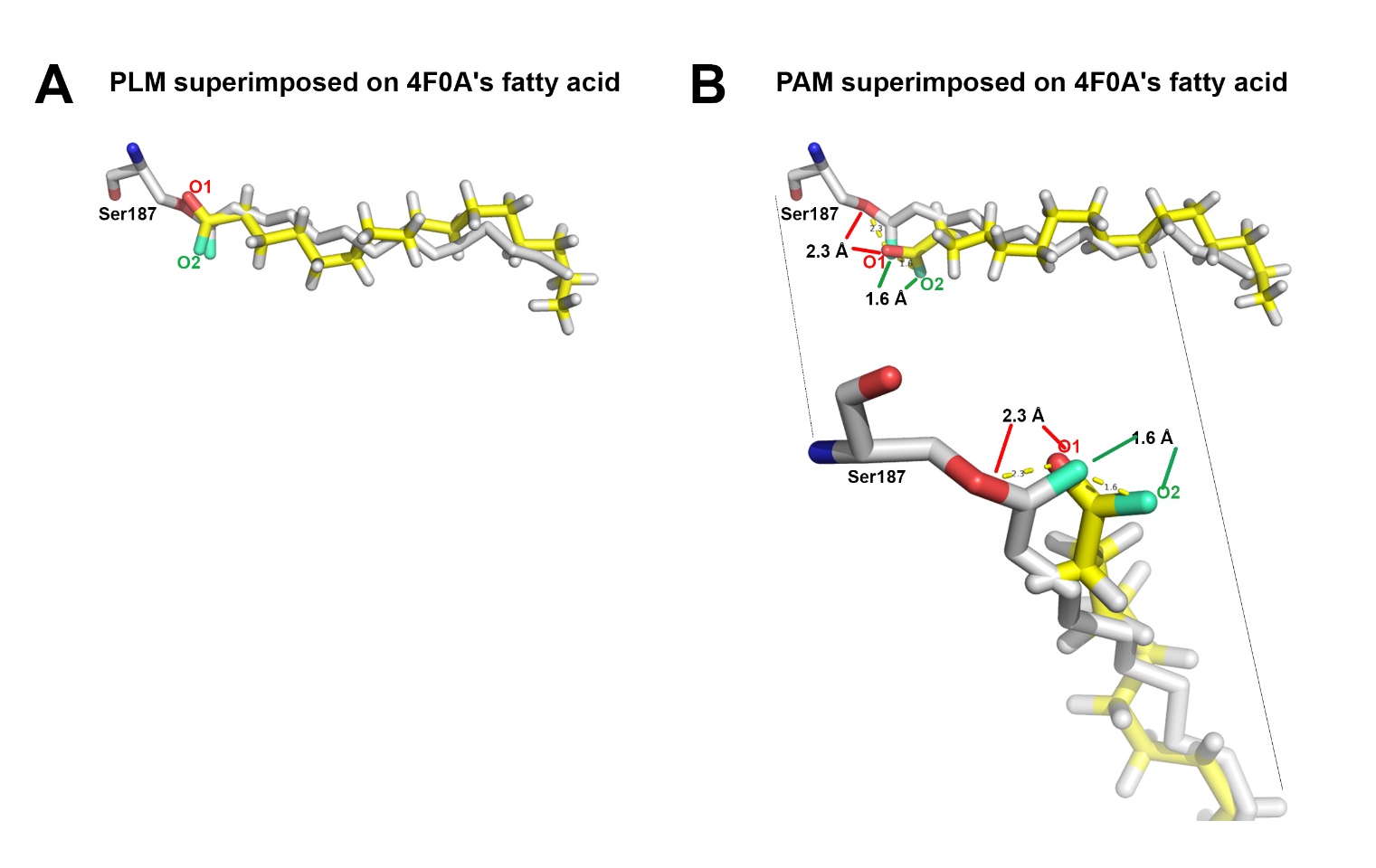
 **Figure S3: PLM's O2 atoms is in the optimal orientation to be covalently attached to the target serine residue.** In both panels, 4F0A's fatty acid covalently bound to Ser187 of xWnt8 is depicted in gray sticks, while our modeled ligands are depicted in yellow sticks. **(**A) PLM's O1 and O2 oxygen atoms fit perfectly to the corresponding reference oxygen atoms. **(**B) On the contrary, O1 and O2 of PAM are 2.3 Å and 1.6 Å away from their reference values, respectively.


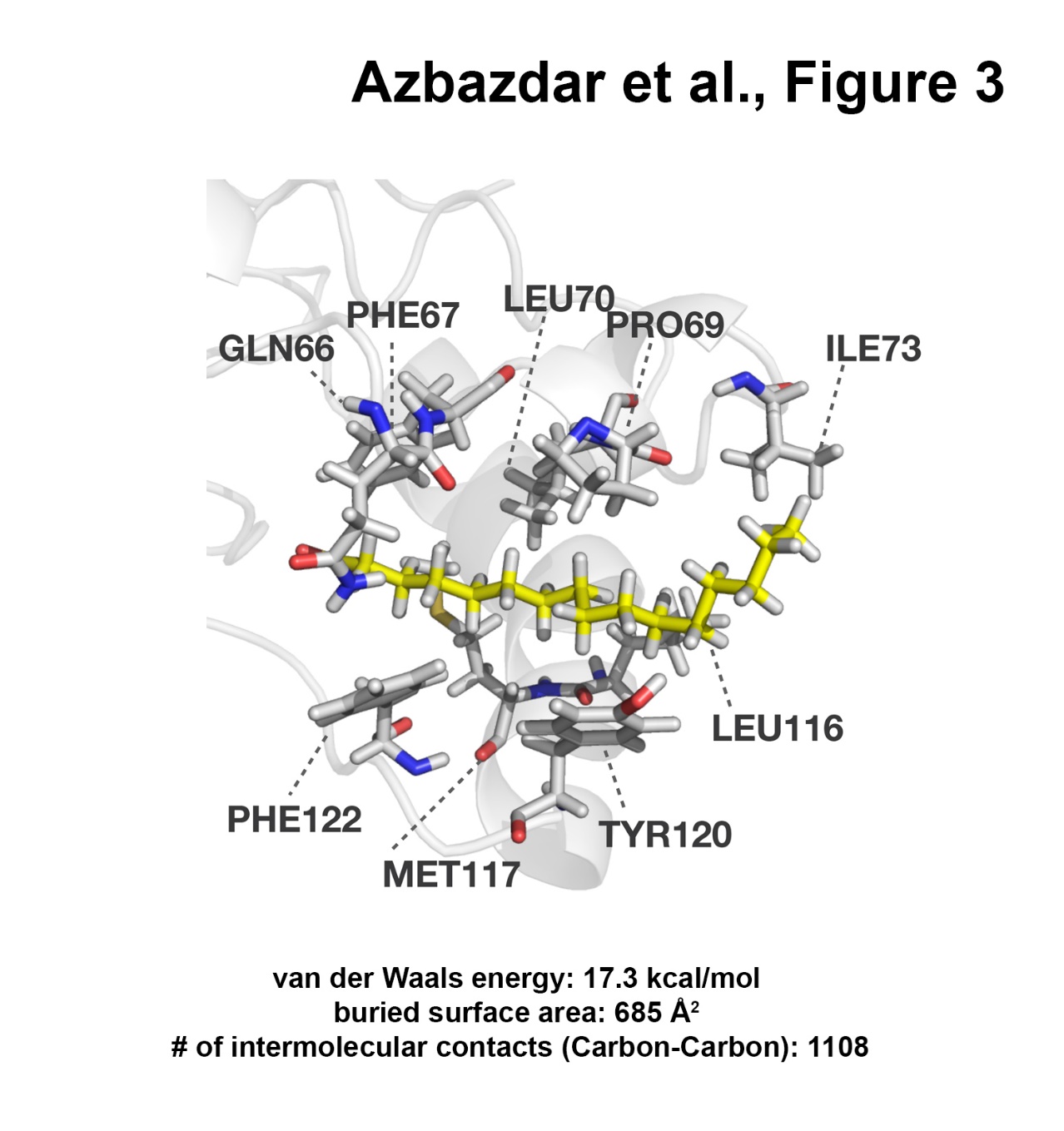


**Figure S4. Modeled Zebrafish Fz8 interactions with PLM.** Our zebrafish model of Fz8-PLM suggests that the PLM molecule interacts with Fz8’s GLN66, PHE67, PRO 69, LEU70, ILE73, LEU116, MET117, TYR120, PHE122 amino acids.

**
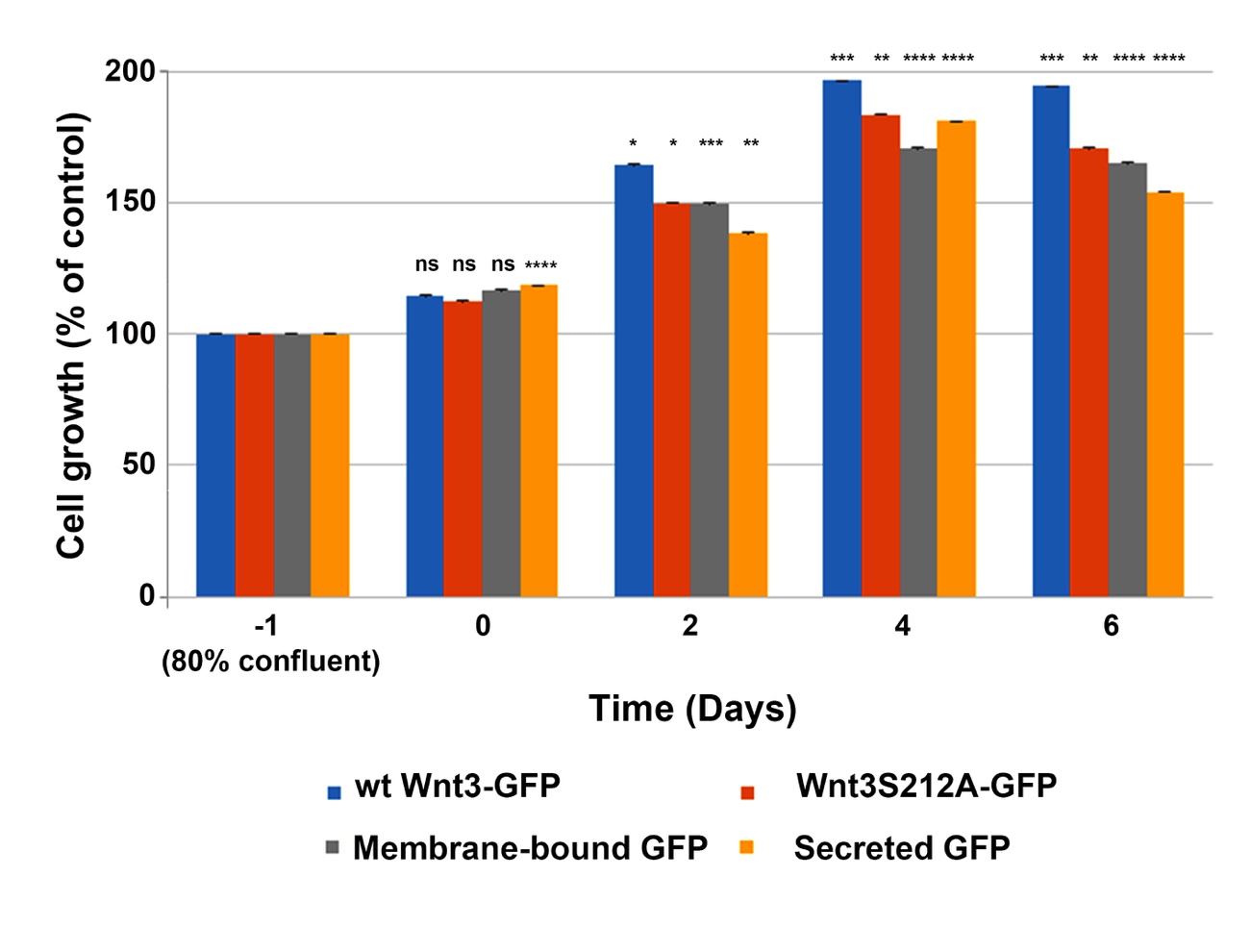
**

**Figure S5.** Cell growth evaluated by MTT assay. HEK293T cells transfected with either of the plasmids (wt Wnt3-GFP, Wnt3S212A-GFP, membrane-bound GFP or secreted GFP) are viable at 80% confluence (day -1) and continue to grow through days 0, 2, 4 and 6. Statistical significance was evaluated using unpaired t-test. **** indicates p < 0.0001, *** p < 0.001, ** p < 0.01, * p < 0.05 and ns nonsignificant. Error bars represent SD.


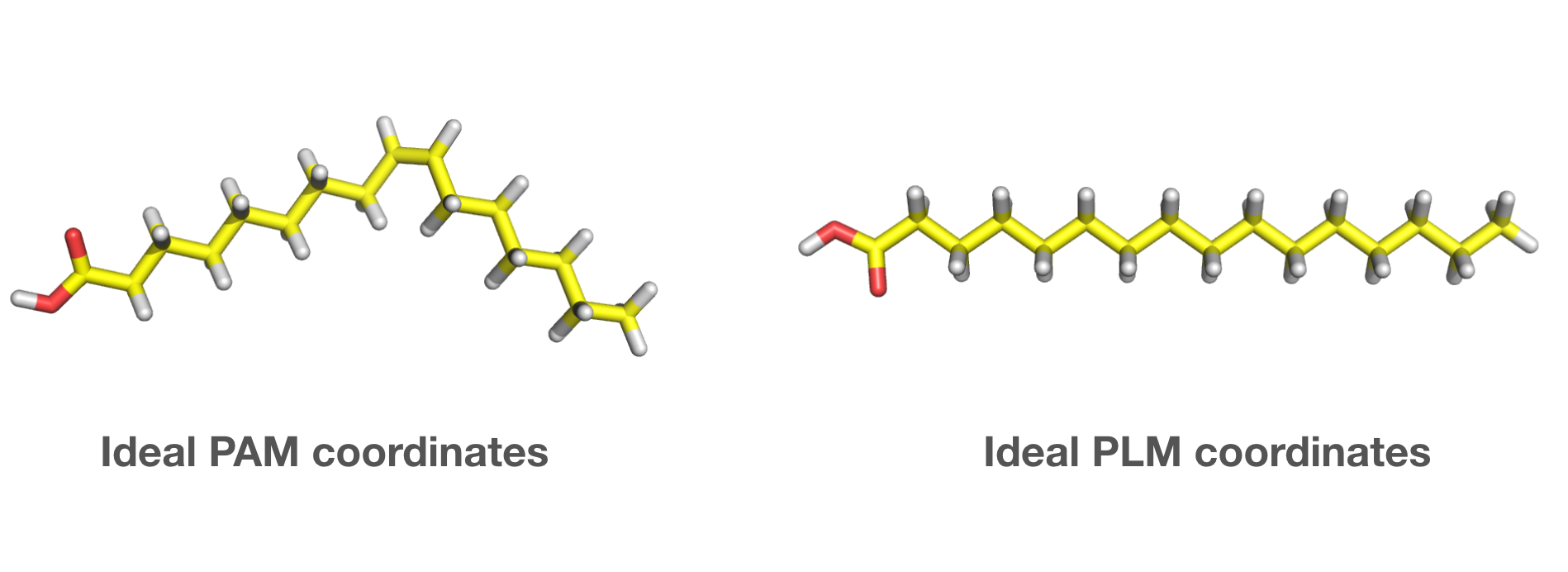


**Figure S6.** PAM and PLM ligand topologies were generated by using their ideal geometries deposited in http://ligand-expo.rcsb.org. Then their CNS parameters were calculated with Prodrg.
